# Flexible, stretchable, on-chip optical tweezers for high-throughput bioparticle manipulation

**DOI:** 10.1038/s41377-026-02199-4

**Published:** 2026-02-03

**Authors:** Ziyi He, Jianyun Xiong, Yang Shi, Ting Pan, Shaobiao Chen, Xin Zhang, Yizhen Chen, Xiangxian Wang, Baojun Li, Hongbao Xin

**Affiliations:** 1 Guangdong Provincial Key Laboratory of Nanophotonic Manipulation, Institute of Nanophotonics, College of Physics & Optoelectronic Engineering, Jinan University, Guangzhou, China; 2 School of Science, Lanzhou University of Technology, Lanzhou, China

**Keywords:** Optical manipulation and tweezers, Optical physics

## Abstract

High-throughput trapping and precision manipulation of individual pathogenic bioparticles in complex microenvironments are of great importance for in-vitro diagnostics and drug screening. Although optical tweezers have been widely used for bioparticle trapping and manipulation, the throughput, functionality, and adaptability are still limited for on-chip integrated bioparticle manipulation in complex and dynamic bioenvironments. Here, we report flexible, stretchable, on-chip optical tweezers (FSOT) based on large-scale orderly assembled microlenses for high-throughput manipulation of bioparticles in complex bio-environments and on flexible substrates, including soft bio-substrates such as skin and intestines. Large-scale (up to 1000) photonic nanojet effect of the microlenses enables high-throughput trapping, sorting, and modulation of individual bioparticles with sizes ranging from sub-100 nm to tens of micrometers, such as exosomes, bacteria and mammalian cells. Our FSOT exhibits high flexibility, which enables bioparticle trapping and sorting in complex and curved biological microenvironments. Importantly, our FSOT also exhibits high deformability and stretchability, which facilitates the control of inter-cellular distance between trapped neighboring cells, enabling real-time modulating and monitoring the interaction between single pathogenic bacteria and macrophage. Our FSOT represents a new class of on-chip optical tweezers for high-throughput bioparticle trapping and manipulation with the features of high flexibility and stretchability, and holds great promises as an integrated on-chip platform for high-throughput dynamic analysis of bioparticles, for revealing inter-cellular interactions between pathogenic bioparticles and host cells, and for precise drug screening.

## Abstract (web summary)

We developed flexible, stretchable, on-chip optical tweezers (FSOT) capable of high-throughput trapping and manipulation of bioparticles with sizes ranging from sub-100 nm to tens of micrometers on curved bio-substrates such as skin and intestines.

## Graphical abstract

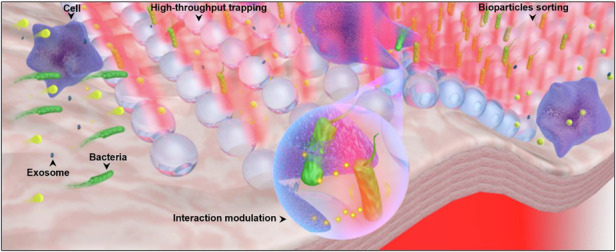

## Introduction

Recent advances in biomedical research have highlighted the critical role of precision bioparticle analysis in pathogen detection, disease diagnostics, drug screening, and therapeutic development^1–3^. Tiny biological particles, such as bacteria, viruses, and exosomes, can carry early signs of disease or act as potential disease-transmission vectors^4–7^. Such pathogenic bioparticles widely exist in different complex microenvironments. Consequently, multifunctional and high-throughput manipulation, such as capture^8,9^, sorting^10–13^, and interaction modulation^14–16^, of different pathogenic bioparticles in complex microenvironments is of great importance for precision bioparticle analysis and will further facilitate our better understanding of the pathogenic and inter-cellular interaction mechanisms of different bioparticles at different living states^17–19^.

To achieve stable trapping and manipulation of bioparticles, a variety of capture platforms have recently been developed^20–22^. Among these methods, techniques utilizing external fields are commonly employed to apply controlled forces for stable particle capture. For example, standing-wave-based acoustic tweezers can trap bioparticles in fixed positions, enabling diverse patterning of captured particles with high throughput^23–25^. Optoelectronic tweezers use a combination of dielectrophoresis and optical regulated micro-well arrays to enhance large-scale trapping and dynamic sorting^26–28^. Magnetic tweezers leverage specific interactions between bioparticles and receptors attached to magnetic beads to achieve selective trapping^29–31^. Although these methods enable particle trapping and manipulation, they face inherent challenges in spatial resolution and biocompatibility for nanoscale bioparticle trapping. Alternatively, conventional optical tweezers (COTs) based on a highly focused laser beam demonstrate exceptional precision in non-invasive particle trapping and manipulation with minimal damage, and have been widely used for bioparticle trapping and manipulation^32–36^. However, the throughput for bioparticle manipulation is limited by the single focused beam. Although the development of holographic optical tweezers (HOTs) can be used for large-scale particle trapping using modulated holographic patterns, which greatly increases the manipulation throughput^37,38^, due to the diffraction limit, the trapping and manipulation of nanoscale bioparticles is very difficult. In addition, both COTs and HOTs face significant challenges for on-chip integration. Such on-chip integration is very important for bioparticle manipulation and analysis in a highly compact and broadly accessible manner in complex microenvironments. To address these limitations, different integrated on-chip optical tweezers platforms have been emerged based on silicon^39^ or polymer waveguides^40^, plasmonic nanostructures^41^, and all-dielectric metasurface^42^. Among them, the nanoplasmonic based on-chip platforms are very useful for the trapping of nanoscale bioparticles due to the excitation of hotspots much smaller than wavelength^43^. Despite these advancements, the above-mentioned on-chip optical trapping platforms predominantly employ rigid substrates, which severely limit their compatibility and adaptability with curved biological surfaces and dynamic microenvironments^44,45^. Nevertheless, many of the bioparticles are in complex dynamic environments with different structured morphology, and different bioparticles are in dynamic processes with inter-cellular communications between neighboring or host cells. Although the existing on-chip integrated optical trapping platforms can be used for in vitro settings, the lack of mechanical adaptability limits the further use for bioparticle manipulation and analysis in the complex microenvironment with potential integration of other emerging applications in implantable sensors and wearable diagnostics^45^.

**Fig. 1:**
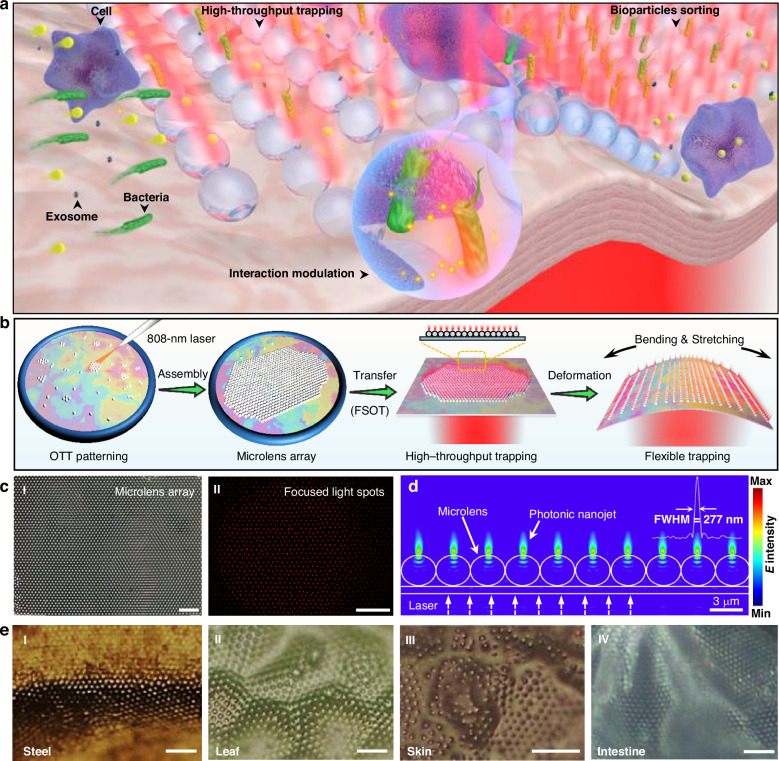
Fabrication of FSOT. **a** Schematic diagram of FSOT for high-throughput bioparticle manipulation on biological substrates. The FSOT can be used for trapping, sorting, and inter-cellular interaction modulation of different bioparticles in a flowing environment. **b** Schematic diagram of the fabrication process of OTT-mediated FSOT system. **c** Panel I: microscopic image showing assembled large-scale microlens array. Panel II: darkfield image showing the corresponding focused light spots. **d** Simulated light distribution of the photonic nanojet array. The inset shows the FWHM of the light intensity at the focal planes of the photonic nanojet in the *Y* direction. **e** Microscopic images of assembled large-scale microlens array on (I) steel tube, (II) leaf, (III) skin, and (IV) intestine. Scale bars: 20 μm

To address these limitations, in this work, for the first time, we report flexible, stretchable, high-throughput on-chip optical tweezers (FSOT) for high-throughput bioparticle manipulation in complex biological microenvironments. Our FSOT is fabricated based on orderly assembled large-scale microlenses on different flexible substrates for bioparticle trapping. The photonic nanojet effect generated by the microlens array enables the generation of up to 1000 sub-wavelength light focuses, which enables high-throughput trapping and manipulation of different bioparticles with sizes ranging from sub-100 nm to tens of micrometers, such as exosomes, bacteria, mammalian cells (Fig. 1a). The high flexibility and stretchability of the substrate allow the FSOT to perfectly adapt to complex curved surfaces and even biological tissues for bioparticle trapping in integrated microfluidic and in vitro settings. The flexibility of our FSOT also enables effective sorting of different sized bioparticles either on flat or curved substrates due to the different optical forces. Additionally, the stretchability enables us to control the distance between neighboring trapping sites, and thus to control the inter-cellular distance between trapped neighboring cells. This capability facilitates real-time modulation and monitoring of inter-cellular interactions such as immune responses between pathogenic bacteria and immune cells.

## Results

### Fabrication of FSOT

The core part of our FSOT is the orderly assembled large-scale microlenses with sub-wavelength light focusing capability, i.e., photonic nanojet effect^46^, on flexible substrates. The assembly of such orderly microlenses array requires the use of a flexible and adaptable assembly carrier. Soap films, with their unique properties of high flexibility and deformability, serve as an excellent candidate for particle assembly^47,48^. To fabricate the FSOT, soap films were chosen as the carrier for assembling orderly microlens arrays via opto-thermal-tension (OTT) effect. During the assembly process (Fig. 1b), titanium dioxide (TiO₂) microspheres were randomly dispersed on the soap film with a thickness of 100–200 nm (details see Methods Section). A laser beam with a wavelength of 808 nm was then directed onto the soap film through a tapered optical fiber probe fixed on a micro-manipulator (details see Methods Section). This 808 nm laser beam caused an opto-thermal effect on the soap film’s surface, altering its surface tension, causing the OTT effect. Through the OTT effect, precise control of the microspheres on the soap film was achieved, allowing them to be moved (with a resolution of 450 nm), rotated (with a resolution of 1 rad), and finally re-ordered). The OTT effect ultimately enabled the assembly of a large-scale microlens array with thousands of closely packed and orderly arranged microspheres, on the flexible soap film (details of the OTT method see Fig. S1). Due to the flexible, deformable and adhesive properties of the soap film, and to increase the stability of the assembled microlenses and to finally form the FSOT, the microlens array was then transferred from the soap film to a flexible substrate polydimethylsiloxane (PDMS) (details see Methods Section). Figure 1cI shows an example of tightly packed and orderly TiO₂ microlens (3 μm in diameter) array on a flexible PDMS substrate. Compared to conventional methods that directly fabricate microlens arrays on target substrates, such as inkjet printing^49^, nanoimprint lithography^50^, and photolithographic thermal reflow^51^, by flexible transferring the assembled microlens array from soap film to different substrates, our method exhibits excellent compatibility with curved surfaces with complex morphology, such as concave, convex, or even sharp-edged substrates.

A 980-nm laser beam output from a multi-mode optical fiber probe was directed perpendicular to the PDMS substrate. This irradiated laser beam was then re-distributed and focused by each microlens, forming up to 1000 focused light spots. As shown in Fig. 1cII, for better visualization, a red laser was employed to illustrate the focusing effect of the microlens array via the photonic nanojet effect of the microlenses. It should be noted that the number of the focused light spots depends on the light irradiation area from the fiber probe, and can be further increased by increasing this irradiation area with a free space light irradiation. The light focusing effect by the microlens can generate a sub-diffraction-limited photonic nanojet and can be used for trapping of nanoscale particles. Two key factors influence this ability: the refractive index (*n*) and the diameter (*D*) of the microlens. To analyze this ability, numerical simulations were conducted (Fig. S2). It was found that compared to polystyrene (PS) microlenses (*n* = 1.59) and silicon dioxiede (SiO₂) microlenses (*n* = 1.47), titanium dioxide (TiO₂) microlenses (*n* = 2.6) generated the strongest focusing effect, with a maximum light intensity of 1.51 and 2.18 times to that of PS and SiO₂ microlenses with the same diameter of 3 μm. Furthermore, simulations with TiO₂ microlenses of 2, 3, and 4 μm in diameter revealed that the light focusing capability of the 3 μm microlenses is the strongest, making them optimal for constructing the FSOT. Therefore, in the experiments, 3 μm TiO₂ microspheres were chosen as the microlenses to form our FSOT. Figure 1d shows the light-field distribution of the photonic nanojets generated by the microlens array (3 μm TiO₂ microspheres), with a 277 nm of full width at the half maximum (FWHM) for the focused light spot.

Importantly, due to the high flexibility of the PDMS substrate, even when the flexible substrate was deformed, such as bending and stretching, the assembled microlens array can keep stable, and the light focusing capability can be maintained (Fig. S3). Therefore, flexible, stretchable, on-chip optical tweezers (FSOT) was formed. In particular, due to the deformable and adhesive properties of the flexible soap film, the assembled large-scale microlens array can also be transferred to many other different complex functional substrates with irregular morphology, both hard and soft surfaces. As some examples, Figure 1e shows the transferred large-scale microlens array on curved hard steel surfaces, undulating soft leaves, elastic skin and wrinkled intestinal tissue. This capability enables us to form FSOT on different complex substrates for bioparticle manipulation in various on-chip integrated environments and in vitro settings, with the potential to be integrated with other emerging applications in implantable sensors and wearable diagnostics in different in vitro settings.

### High-throughput bioparticle trapping

**Fig. 2:**
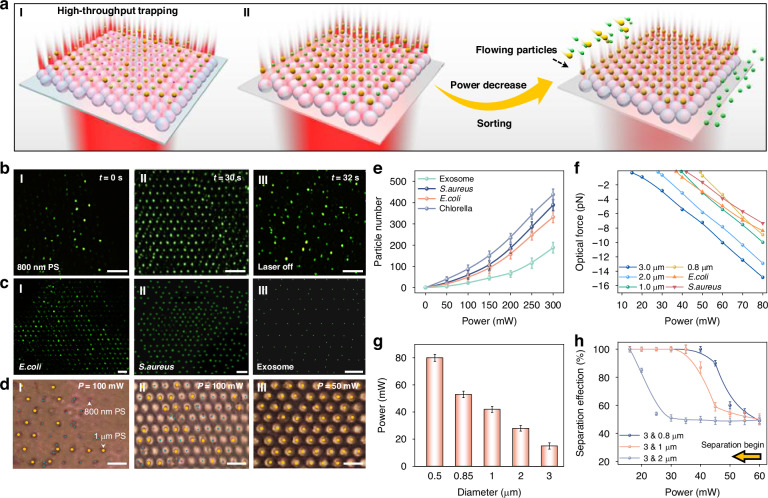
High-throughput bioparticle trapping and sorting. **a** Schematic diagram showing (I) high-throughput trapping and (II) sorting of different bioparticles using FSOT. **b** Microscopic images showing the trapping of 800 nm particles. **c** High-throughput trapping of bionanoparticles such as (I) *E. coli*, (II) *S. aureus* and (III) exosomes. **d** Sorting of 800 nm (blue dots) and 1 μm (yellow dots) PS particles. **e** Number of trapped bioparticles as a function of the laser power. **f** Calculated optical force exerted on particles as a function of laser power. **g** The laser power threshold for particle trapping as a function of particle size. **h** Sorting efficiency as a function of the capture laser power during the separation process. Scale bar: 5 μm

Different from previous single photonic nanojet-based nanoparticle trapping^52^, the large-scale photonic nanojets of our FSOT greatly increase the throughput of trapping and manipulation of different bioparticles (Fig. 2a). Due to the different forces required for stable trapping of particles with different sizes, by changing the optical power, particles with different sizes can thus be sorted. For high-throughput bioparticle manipulation and sorting, we constructed the FSOT on a microfluidic platform (Fig. S4, details in the Methods Section). To validate the high-throughput trapping capability of the FSOT, experiments were conducted on particles of various sizes. As shown in Fig. 2b, 800 nm PS nanoparticles in green fluorescent were randomly distributed without laser irradiation. Upon applying the 980-nm trapping light (300 mW) for 30 s, the nanoparticles were orderly trapped at the focal points of the microlens array, with up to 327 particles captured (Fig. 2bII). With the light off, the trapped particles were released (Fig. 2bIII). Our FSOT also enables the high-throughput trapping of particles with different sizes, including those far below the diffraction limit. Fig. S5 shows the high-throughput trapping of PS particles with different sizes ranging from sub-100 nm (95 nm) to 2 μm, with capture counts up to 400 for 2 μm particles (Fig. S5c). Importantly, the FSOT can also successfully trap biological particles, including *E. coli*, *S. aureus*, exosomes, and Chlorella algae, with particle counts of about 389, 332, 188, and 438, respectively (Fig. 2c, e and Fig. S6). These capabilities are essential for further precision and high-throughput analysis of bioparticles in microenvironments.

To further analyze the trapping capability of our FSOT, we numerically calculated the optical force exerted on different particles based on 3 μm TiO_2_ microlenses. Figure 2f shows the optical forces exerted on particles with different sizes, and the force is linearly related to the laser power. For stable trapping in a flowing fluid, the optical force exerted on particles should overcome both Brownian motion and fluidic drag force. As a result, for stable trapping of different particles, a specific threshold of optical power is required to generate an effective optical force to overcome fluidic drag force and Brownian motion (Fig. 2g). The power threshold is closely related to particle size. The optical gradient force exerted on the particles is cubically increased with the radius *r*, while the fluidic drag force is linearly increased with particle size. The cubic growth of optical gradient forces results in greater trapping force for larger particles in flow environments. In contrast, smaller particles generally require significantly higher capture power thresholds. This is because the optical gradient force exerted on smaller particles increases slowly and their Brownian motion is much stronger, and thus higher laser power is required to compensate for the capture. This feature indicates that our FSOT is capable of effective sorting and separation of various particles by changing the laser power. To confirm this sorting capability, experiments based on PS particles with different sizes were carried out. As shown in Fig. 2d, with optical power of 100 mW, both 800 nm and 1 μm particles were trapped with an amount of 103 and 108, respectively (Fig. 2dI-II). By gradually decreasing the power, the 800 nm particles were gradually released due to the weaker trapping force. As the capture power was decreased to 50 mW which was lower than the capture power threshold of the 800 nm particles, the difference in optical force exerted on these two is 2.4 pN. The 800 nm particles were completely released, while the 1 μm particles were still trapped. Therefore, selective trapping and sorting of these two different-sized particles was realized (Fig. 2dIII and Fig. S7). This method is also applicable for the sorting of particles with other sizes as shown in Fig. 2h.

### Flexibility of FSOT for particle trapping/sorting on curved substrates

**Fig. 3:**
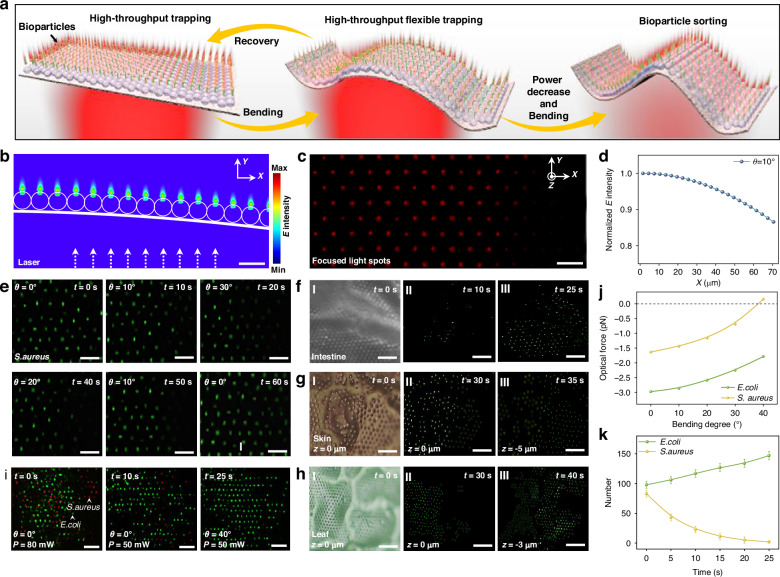
Flexibility of FSOT for particle trapping/sorting on curved substrates. **a** Schematic diagram showing flexible trapping and sorting of bioparticles on curved substrates. **b** Simulated light distribution of photonic nanojet on 10° bent substrate. **c** Dark-field images showing focused light spots above the bent substrate. **d** Normalized light intensity on 10° bent substrate. **e** Microscopic images showing the stable capture of *S. aureus* (green) during reciprocating bending. **f**–**h** Microscopic images showing the capture of exosomes (green) on (**f**) intestine tissue, (**g**) skin, and (**h**) leaf with different focus height *z*. **i** Microscopic images showing sorting of *E. coli* (green) and *S. aureus* (red) by changing the laser power and bending angle of the FSOT. **j** Calculated optical force exerted on *E. coli* and *S. aureus* as a function of the bending angle. **k** Number of trapped *E. coli* and *S. aureus* as a function of time during the FSOT bending process. Scale bar: 5 μm

In the real biomedical settings, bioparticles are generally in complicated environments with complex morphology. The ability of bioparticle trapping in such complicated environments is very important for further precision in situ analysis. Our FSOT not only enables high-throughput bioparticle trapping and manipulation on a flat and smooth substrate, but also exhibits excellent flexibility with remarkable capabilities for bioparticle trapping and manipulation on curved substrates and even on biological tissues (Fig. 3a). Due to the high flexibility of the PDMS substrate, our FSOT can be flexibly bent (details see Methods Section). After the bending, the assembled microlenses remain stable, and the incident light can also be focused. To investigate the light-field distribution via photonic nanojet effect of microlenses on curved substrate, numerical simulations were first conducted (Fig. 3b). The simulation revealed that while the curved microlens array can effectively focus the incident laser beams, the reflection and refraction of light at the air-substrate medium interface reduce the intensity of the transmitted light. According to the Fresnel equations, this intensity loss is dependent on the bending angle of the substrate, as a larger bending angle causes more reflection loss in transmitted light (Fig. 3d). Consequently, as shown in Fig. 3c, the brightness of the focused light spots formed by the curved microlens array decreases progressively with the increased substrate bending angle. As shown in Fig. S9, when the bending angles are 20°, 30°, and 40°, the optical field intensity at the free end decreases to 0.75, 0.65, and 0.56 of that at the fixed end, respectively. However, the array maintains its ability to stably trap particles of various sizes due to the light focusing capability of the microlenses. To validate these theoretical predictions, experiments were conducted on curved substrates with different bending angles. Experimental results show that high-throughput particle trapping is capable even with bending angle up to 40°, but the number of trapped particles is decreased with the increase in bending angle. To further demonstrate the trapping performance of the FSOT with different bending angles, we performed additional trapping experiments using 1 µm PS particles with different powers by bending the substrates with different angles (Fig. S11a–c). As shown in Fig. 2g, when the substrate was flat (0°), the trapping power threshold was 42 mW. As the bending angle *θ* was increased from 0° to 10°, 20°, 30°, and 40°, the threshold was gradually increased from 42 to 72, 118, 180, and 250 mW, respectively (Fig. S11d). Beyond 40°, stable trapping could not be achieved. To effectively mitigate the optical loss between the substrate and the microlenses, a potential strategy by inserting an index-matching layer between them can be considered (Fig.S12a). As shown in Fig.S12b–d, simulation results indicate that a matching layer with a refractive index of 1.8 and a thickness of 170 nm between the PDMS substrate and the TiO2 microlens can result in a best choice with the optical transmission efficiency increased by 125%. Details of the trapping and stability analysis can be seen in Figs. S8–13. In general, the optical trapping force is affected by bending angle. With the increase of the bending angle, the optical trapping force is decreased. These results indicate that our FSOT enables high-throughput particle trapping in environments with complex morphology and bent substrate, but the trapping stability is decreased with the increase in bending angle, and the trapping failed in highly curved regions (*θ* > 40°).

The high throughput trapping capability of the FSOT at bending state is also applicable for bioparticles with different sizes. As shown in Fig. 3e, when *θ* = 0°, about 80 green-fluorescent *S. aureus* were trapped with an optical power of 100 mW. Subsequently, the substrate was gradually bent to 20°, 30°, and then recovered back to 20°, 10°, and finally returned to *θ* = 0°. Throughout this bending process, despite the constant deformation of the flexible substrate, *S. aureus* maintained trapped with an orderly arrangement. These results demonstrate the stable trapping capability during the deformation process. To further demonstrate the bioparticle trapping capability on the curved FSOT, other different sized bioparticles, such as rod-shaped *E. coli*, 3-μm Chlorella algae, have also been stably trapped during the bending process, as shown in Fig. S14.

In addition to bending with a controlled angle, our FSOT can also be applied for substrates with more complicated morphology, which can be found in real in-vitro settings. To show such capability, experiments were carried out by forming FSOT on different biological substrates, for example, intestines, skin, and leaf. In particular, we used exosomes (about 100 nm in size), which are very important bioparticles with increasing research interests for disease detection and therapeutics, for bioparticle trapping using flexible FSOT formed on such complicated bio-substrates. As shown in Fig. 3f, after transferred to the surface of an intestine tissue, the microlens array was flexibly adjusted to the arrangement according to the folds of the intestine. With an optical power of 150 mW, about 200 exosomes in green fluorescence were successfully captured in 60 s. To further show the high-throughput trapping capability on curved bio-substrate, trapping of exosomes at different regions of rough skin and soft leaves with different heights (*z*) was successfully achieved (Fig. 3g, h and Fig. S15). Further experiments indicate that the structure and trapping capability of our FSOT on living tissue environment (such as intestinal tissues) remains stable during the prolonged operation with a duration up to 120 min (Figs.S16 and S17). During the prolonged trapping process, no obvious photothermal damage to the intestinal tissues and trapped bacteria were observed with a duration of 120 min (optical power: 250 mW).

The ability to selectively trap specific bioparticles in complex environments is crucial for precision bioparticle/drug screening and analysis. Importantly, with different bending angles of the substrate, the optical force exerted on different bioparticles is different, enabling precise separation of different bioparticles using our flexible FSOT. For instance, as demonstrated in Fig. 3i, with an optical power of 100 mW applied on a flat FSOT, both red-fluorescence *S. aureus* and green-fluorescence *E. coli* were simultaneously trapped with a number of 83 and 98 respectively. Calculation results indicate that the optical forces exerted on *E. coli* and *S. aureus* are different with different bending angle of the FSOT (Fig. 3j). In particular, as the substrate is bent to 40°, the force exerted on *S. aureus* becomes positive, indicating a repelling force, while that on *E. coli* is still a trapping force of 1.8 pN, and thus the two kinds of bacteria can be separated. In the experiments, the substrate was deformed with bending angle of 40°, the red-fluorescence *S. aureus* were gradually released due to the repelling force, achieving the selective trapping of *E. coli*. At *t* = 25 s, the sorting efficiency was 100% (Fig. 3k). It should be noted that, while the reduction in trapping force generally weakens the trapping capacity for both types of bacteria, the force exerted on rod-shaped *E. coli* is stronger than that on *S. aureus*, which remains above the threshold for effective trapping of *E. coli*. Consequently, as *S. aureus* escapes from the trapping by fluidic flow, *E. coli* in the flowing environment can still be stably trapped when passing over the trapping positions. This ultimately leads to an increase in the number of *E. coli* during the separation process. Compared with the sorting using a flat FSOT by adjusting the optical power to generate a different value of optical trapping force, as shown in Fig. 2f, bending of the FSOT can lead to the generation of optical forces with different directions, i.e., repelling and trapping force, which is more efficient for sorting of particles with different shapes and sizes. This capability highlights the potential of our FSOT for the selective trapping and sorting of different bioparticles in complex microenvironments for further precision analysis.

### Stretchability of FSOT for inter-cellular interaction modulation

**Fig. 4:**
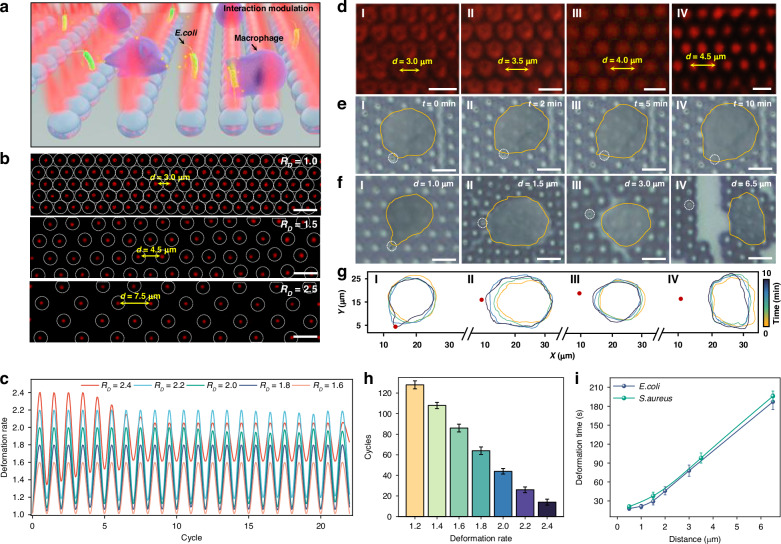
Stretchability of FSOT for inter-cellular interaction modulation. **a** Schematic diagram showing modulation of inter-cellular interaction between *E. coli* and macrophage by stretching of the FSOT. **b** Dark-field images showing the stretching of FSOT with deformation rate (*D*_R_) of (I) 1.0, (II) 1.5, and (III) 2.5. **c** Stretching recovery capacity of FSOT system as a function of deformation cycles. **d** Microscopic images showing capture of Chlorella during FSOT stretching. **e** Microscopic images showing the inter-cellular interaction between *E. coli* and macrophage on FSOT with an initial distance of 500 nm between the macrophage and the *E. coli*. Yellow curve represents the boundary of the macrophage, and white dashed circle represents *E. coli*. **f** Microscopic images showing the interaction between macrophage and *E. coli* for 10 min with different inter-cellular distances. **g** Real-time boundary of the macrophage during inter-cellular interaction at different initial interaction distances. The initial distances between macrophage and *E. coli* in images I-IV are (I) 1, (II) 1.5, (III) 3, and (IV) 6.5 μm, respectively. **h** Recoverable deformation cycles of FSOT as a function of the deformation rate. **i** The deformation time of macrophages as a function of the distance between different bacteria controlled by the FSOT. Scale bar: 5 μm

Precision modulation of inter-cellular distance is crucial for better understanding inter-cellular interaction and communication. Although different optical trapping platforms can be used to trap different bioparticles, previous platforms are difficult to control and modulate the inter-cellular interactions. In addition to being bendable, the high flexibility of our FSOT also exhibits excellent stretchability. This enables the precise control of the distance between neighboring trap positions, and further allowing the modulation of inter-cellular interactions of trapped bioparticles, such as immune response between macrophages and pathogenic bacteria (Fig. 4a). To show the stretchability of our FSOT, the substrate with microlenses orderly assembled was stretched (details see Methods Section). The deformation rate of the original microlens array without stretching was defined as *R*_D_ = 1.0. As shown in Fig. 4b, the microlens array was stretched with *R*_D_ of 1.0, 1.5, and 2.5, respectively. The size and light intensity of the focused light spots remained consistent during the stretching and deformation, indicating that the FSOT’s trapping performance was well maintained. We also tested the stretching repeatability of the FSOT by stretching it back and forth with different *R*_D_ (Fig. 4c). We found that with a deformation rate less than 2.0, the recover capability of the FSOT remained quite well with up to 50 cycles of stretching (Fig. 4h). Such recover capability can even remain for 130 cycles of stretching with an *R*_D_ less than 1.2. However, the recovery capability was destroyed after 4 cycles of stretching with an *R*_D_ of 2.4. This stretchability and recoverability also exist when stretching along other directions. For example, as shown in Fig. S18a, b, after the microlens array was stretched with deformation rate of 2.0 and 4.0 under uniaxial (perpendicular) and biaxial (both perpendicular and parallel) direction, respectively, the array remained stable. During the stretching, for the deformation rate within 2.0, the microlens array exhibits high recoverability even after 20 cycles of stretching (Fig. S18c).

This stretchability and recoverability of the FSOT enables us to control the interaction distance between neighboring bioparticles. First, we tested this capability by capturing red fluorescence Chlorella cells. When stretching the FSOT with a *R*_D_ up to 1.5, the Chlorella remained stably trapped during the stretching, and their intercellular distances were adjusted accordingly (Fig. 4d). These results demonstrated that our FOST could precisely control the interaction distance between neighboring bioparticles.

Leveraging this control, we then investigated the capability to modulate the inter-cellular interaction and immune response between single macrophage and pathogenic bacteria using our FSOT. Macrophages are essential for recognizing, sensing, and killing pathogenic bacteria. Their surfaces present multiple signal receptors that can detect signal factors released by bacteria, thus regulating the process of phagocytosis. This signal recognition can also promote actin assembly and cytoskeleton remodeling of macrophages with subsequent pseudopodia formation, and macrophages can then deform and move toward bacteria. By stretching the FSOT, we controlled the distance between a trapped *E. coli* bacterium and a macrophage. This process enables macrophages to identify bacteria at varying distances and to elicit distinct immune responses. First, a *E. coli* was captured with an interaction distance of only 500 nm to a macrophage (Fig. 4e). When the signal molecules released by *E. coli* were detected by macrophage receptors, the macrophage became activated and deformed toward *E. coli* within two minutes. The *E. coli* was phagocytosed by the macrophage after 10 min. We further investigated the immune interaction between *E. coli* and macrophage by stretching the FSOT with different interaction distances of 1, 1.5, 3, and 6.5 μm (Fig. 4f). The morphological changes of the macrophage during the whole interaction process were shown in Fig. 4g and Fig. S19. We found that the time for the macrophage to start deforming after recognizing the *E. coli* was increased with the increase in distance. As the distance between the macrophage and *E. coli* increased, the macrophage’s deformation slowed down with decreased pseudopodia growth. This is probably because the concentration of detectable signal molecules released by *E. coli* was decreased as the interaction distance increased. Additionally, we also conducted similar experiments with macrophages and *S. aureus* at different distances (Fig. S20). By comparing the response times and morphological changes of macrophages to *E. coli* and *S. aureus*, differences in their response sensitivity were observed (Fig. 4i). These variations could be attributed to differences in receptor affinity for the bacteria’s signal molecules or variations in corresponding receptor distributions on the macrophage cell membrane. Our findings indicate that our FSOT can serve as a platform for direct control, modulation, and observation the interactions between different individual cells.

## Discussion

In summary, we developed flexible, stretchable, on-chip optical tweezers (FSOT) capable of high-throughput and multifunctional bioparticle manipulation in complex environments. Such FSOT was initially constructed using the opto-thermal-tension (OTT) mediated manipulation and assembly of large-scale microlenses on soap films, which were then flexibly transferred onto various complex substrates. The photonic nanojet effect of the assembled microlenses enables the generation of up to 1000 sub-wavelength light focuses. This phenomenon allows for the high-throughput trapping and manipulation of different bioparticles, such as microscale Chlorella algae, sub-microscale bacteria, and nanoscale exosomes. Our FSOT exhibits high flexibility, enabling it to be bent and deformed to adapt to biological substrates with different morphologies, such as intestine tissue, skin, leaves, for bioparticle manipulation. Moreover, by adjusting the trapping power or bending angle, which alters optical force, different bioparticles can be selectively trapped and sorted. Furthermore, our FSOT also exhibits high stretchability, which enables us to precisely control the distance between neighboring trapped bioparticles. This capability enables us to precise control and real-time monitor the inter-cellular interaction and immune response between single bacteria and macrophage.

Currently, there are also a few reports working on “soft” optical trapping with the potential for optical manipulation inside living tissues and other complex environments. For example, Leite et al. reported optical trapping using a high-numerical-aperture soft multimode optical fibre^53^, and this method can be applied for the trapping of particles within a turbid cavity and complex environment, the surface of the optical fiber tip is still rigid, and the throughput of trapped microparticles is less than 10. Although “shapeable” optical traps can be easily created using holography^54^, this holographic optical trapping still face challenge for nanoscale particles due to diffraction limit. In addition, these holographic traps are difficult to construct on curved substrate with complex morphology. Our microlens array-based FSOT can easily achieve high throughput trapping with up to several hundred and even thousand traps, and benefitting from the photonic nanojet effect, our FSOT can be used for the trapping of different sized particles down to sub-100 nm. In addition, due to the flexibility and deformability of the soap film, our FSOT exhibits environmental adaptability, and can constructed on curved substrate with complex morphology, such as biological tissues.

Our FSOT represents as a new integrated on-chip optical manipulation platform for high-throughput bioparticle trapping and manipulation in complex biological microenvironments, with huge potentials for high-throughput bioparticle sorting, pathogenic mechanism analysis, inter-cellular interaction studies, and precision drug screening. Compared with other existing bioparticle trapping methods, our FSOT holds the unique feature of superior flexibility, which enables it to adapt to a wider range of complex environments with different morphology for bioparticle manipulation and precision analysis. The stretchability enables us to precision control the distance between neighboring bioparticles, making it a powerful platform to direct modulate and observe the inter-cellular interaction and communications. It holds great promise in various biomedical research, such as large-scale analysis of pathogenic bacteria, exploration of the antibacterial mechanism of macrophages, and regulation of macrophagic activity. Currently, the OTT assembly process relies primarily on serial and manual operations. To improve the assembly efficiency and throughput, parallel laser patterning would be a good choice. Parallel laser patterning can be employed to assemble multiple microlens arrays simultaneously, and the assembled multiple arrays can then be assembled together to create larger arrays by the surface tension, which significantly improves the assembly throughput. Currently, the high-throughput and multifunctional bio-particle manipulation on our FSOT platform relies on manual operation. Future work could advance this platform toward full automation by employing deep learning algorithms to analyze optical trapping signals in real time for automatic identification and classification of bioparticles; integrating high-speed or high-resolution imaging to simultaneously acquire morphological and fluorescence information during manipulation; and developing an automated, programmable stretching device to dynamically adjust the substrate. The integration of these technologies is expected to yield an intelligent and automated high-throughput on-chip optical manipulation system, providing a more efficient and versatile tool for biomedical applications such as single-cell analysis and pathogen detection. The flexibility and stretchability feature of our FSOT also holds great potential for the combination of optical trapping with other emerging applications in implantable sensors and wearable diagnostics in different in vitro settings.

## Materials and Methods

### Preparation of soap film

First, a soap solution was prepared using deionized water and glycerol in a volume ratio of 4:1. Subsequently, sodium lauryl sulfate, dodecyl dimethylamine oxide, alkylamines, and water-soluble lanolin were added to the solution, achieving final concentrations of 11.9, 44.88, 48.24, and 5.3 mM, respectively. The mixed solution was stirred at 100 rpm for 30 s and then drawn up into a syringe. A filter membrane with a pore size of 500 nm (purchased from Fumei Technology, Xiamen, China) was mounted on the syringe. The syringe was slowly pushed to allow the mixed solution to drip out through the filter membrane. This step was performed to thoroughly filter out bubbles generated during shaking and mixing, as well as impurities in the mixed solution. Next, 10 μL of a 3 μm TiO₂ particle solution (concentration: 0.1 mg mL^−1^) was added to 2 mL of the soap solution and thoroughly mixed to obtain a suspension containing 3 μm TiO₂ particles. A rubber ring with a diameter of 2.5 cm (purchased from Haohuan Sealing Products Co., Ltd, Guangdong, China) was placed into the suspension, and the suspension was continuously added until the rubber ring was completely submerged. After an immersion period of 10 s, the rubber ring was slowly pulled out of the suspension, resulting in the formation of a stable soap film in the central part of the rubber ring.

### Assembly of the microlens array

A tapered optical fiber (TOF), enclosed within an iron pipe, was fixed onto an adjustable five-axis micro-manipulator. This manipulator allowed precise control over the TOF’s three-dimensional movement with a resolution of 0.5 µm and its angular orientation with a resolution of 0.5°. A rubber ring containing a soap film was placed on a two-dimensional moving stage, which provided a resolution of 0.5 µm. Using the TOF manipulator, the tip of the TOF was lowered to a position approximately 20 ~ 30 μm above the surface of the soap film, with an inclined angle of 15°. A laser beam with a wavelength of 808 nm, emitted from a continuous wave solid-state laser, was directed into the TOF to adjust and regulate the surface tension on the soap film. By moving the TOF (808 nm laser beam launched, 20 mW) above a target particle or particle cluster in a forward direction, the particle/cluster was manipulated backward. Large-scale particle array was then assembled using this method.

### Transfer of ordered particle arrays onto different substrates

In the experiment, the assembled particle arrays were positioned at the center of the microscope’s field of view. Using a lifting platform, the substrate was gradually raised at a speed of 0.5 mm s^−1^ until it approached the soap film. Upon contact with the soap film, the substrate adhered to the entire layer of the soap film due to its viscosity. As a result, the orderly arranged particle arrays on the soap film were also transferred onto the substrate along with the soap film. This method was similarly employed to achieve the transfer onto other soft substrates. Therefore, FSOT were formed on different substrates.

### Preparation of bacterial suspension

Gram-negative *E. coli* colony was picked with a syringe and transferred into 5 mL of bacterial culture medium (Lysogeny-Broth nutrient solution). Then, the mixture solution was placed in a constant-temperature shaker with a rotation speed of 200 rpm at 37 °C and incubated overnight. The overnight-cultured bacterial solution was centrifuged, the supernatant was removed, and PBS was added for dilution so that a bacterial solution with a concentration of approximately 1.0 × 10^5^ *E. coli* per microliter was obtained. The Gram-positive *S. aureus* solution can be prepared in the same way.

### Simulation and calculation

The simulation and calculation were carried out using the finite-element method in the Radio Frequency module (Electromagnetic Waves, Frequency Domain) of COMSOL Multiphysics 6.1 software. The trapping laser emitted through the optical fiber probe was set as a 980 nm Gaussian beam.

### Particle trapping and manipulation using the FSOT

A flat-end multimode optical fiber probe surrounded by an iron pipe was installed on an adjustable five-axis micro-manipulator for multi-dimensional spatial adjustment. The optical fiber probe could be moved with a resolution of 0.5 μm in three dimensions and the angle could also be adjusted with a resolution of 0.5°. The 980 nm laser from a continuous wave solid-state laser was transmitted into the optical fiber probe through a multimode fiber. Microflow was introduced into the micro-fluidic channel by two micro-fluidic pumps and were made to pass above the microlens array. During the manipulation on a curved or stretching substrate, both sides of the FSOT are respectively clamped and fixed by two fiber holders. To generate a curved FSOT, one holder remains stable while the other moves toward the center with a resolution of 0.1 μm, causing the FSOT to be bent. To stretch the FSOT, two holders were moved outward at the same rate.

## Supplementary information

Supplementary Information for Flexible, stretchable, on-chip optical tweezers for high-throughput bioparticle manipulation

## Data Availability

All data are available in the main text or the supplementary materials.
