# Supplementary material for: Flexible, stretchable, on-chip optical tweezers for high-throughput bioparticle manipulation
Source: Light Sci Appl. 2026 Feb 3;15:102. doi: 10.1038/s41377-026-02199-4 (PMC12868836; doi:10.1038/s41377-026-02199-4)
Supplement: Supplementary file 1 — Supplementary Information for Flexible, stretchable, on-chip optical tweezers for high-throughput bioparticle manipulation [file 41377_2026_2199_MOESM1_ESM.docx]

**Supplementary Information for**

**Flexible, stretchable, on-chip optical tweezers for high-throughput bioparticle manipulation**

Ziyi He^1^, Jianyun Xiong^1^, Yang Shi^1^, Ting Pan^1^, Shaobiao Chen^1^, Xin Zhang^1^, Yizhen Chen^2^, Xiangxian Wang^2^, Baojun Li^1*^, and Hongbao Xin^1*^

^1^Guangdong Provincial Key Laboratory of Nanophotonic Manipulation, Institute of Nanophotonics, Jinan University, Guangzhou 511443, China

^2^School of Science, Lanzhou University of Technology, Lanzhou 730050, China

^*^ To whom correspondence may be addressed

**Email:** baojunli@jnu.edu.cn, [hongbaoxin@jnu.edu.cn](mailto:hongbaoxin@jnu.edu.cn)

This Word file includes:

1. OTT-mediated microlens assembly on soap film.

Fig. S1. Microlens assembly on soap film.

1. Large-scale photonic nanojet effect via different microlenses.

Fig. S2. Simulated light field distributions of microlens arrays.

Fig. S3. Curved and stretched substrate.

1. Experimental setup.

Fig. S4. Schematic diagram of the experimental setup.

1. High-throughput bioparticle trapping/separation on flat substrate.

Fig. S5. High-throughput capture of multi-scale particles.

Fig. S6. High-throughput capture of different bioparticles.

Fig. S7. Particle separation via adjusting the laser power.

1. High-throughput bioparticle trapping/separation on curved substrate.

Fig. S8. Schematic diagram of the bent substrate.

Fig. S9. Simulated light distribution of photonic nanojet on bent FSOT.

Fig. S10. High-throughput capture on bent FSOT.

Fig. S11. Trapping with different bending angles.

Fig. S12. Simulated light distribution of refractive index matching layer.

Fig. S13. Analysis of trapping stiffness on bent FSOT.

Fig. S14. Trapping of Chlorella on bent FSOT.

Fig. S15. Trapping of exosomes on curved biological substrates.

Fig. S16. Microlens array on sheep intestine.

Fig. S17. Long-term capture of *S. aureus* by FSOT on sheep intestines.

Fig. S18. Stretchability and recoverability of microlens array when stretched along different directions.

Fig. S19. Interactions monitoring of between macrophages and *E. coli*.

Fig. S20. Interactions monitoring between macrophages and *S. aureus*.

1. **OTT-mediated microlens assembly on soap film**

During the moving process, the tapered fiber with an 808 nm laser was moved from right to left along the *x*-axis direction above a particle on the soap film. As the tapered fiber scanning process, the optothermal effect at the directly irradiated spot caused the temperature on the left side of the particle to become higher than that on the right side. Due to the elliptical laser spot (major axis: 60 μm, minor axis: 20 μm), the surface tension on the left side of the particle was lower than that on the right. As a result, the particle was pulled toward the right along the x-axis by the surface tension gradient. When the laser was turned off, the temperature gradient on the soap film decreased rapidly, and the corresponding surface tension gradient gradually diminished to zero, eventually bringing the particle to a stop.

During the rotational manipulation, the tapered fiber tip was held stationary above the interference ring surrounding the particle cluster. The cluster was driven to rotate via the Marangoni flow field generated by the nonuniform temperature distribution induced by the optothermal effect. The direction of rotation could be readily controlled by positioning the OTF on the opposite side of the cluster.

By combining precise translation and rotation of both individual particles and particle clusters via the optothermal effect, targeted assembly of particles into designed patterns on the soap film becomes achievable. Furthermore, large-scale particle structures and crystalline arrangements can be constructed by assembling pre-formed particle modules—much like in a game of Tetris. This assembly is controllable. The assembly of microlens array is a dynamic formation process driven by photothermally induced surface tension gradients. When the laser beam from the tapered optical fiber was irradiated on the interference rings of the soap film surrounding the microlens array, the thickness of the soap film was changed due to light-induced photothermal effects, generating a surface tension gradient. By controlling the movement of the optical fiber, the originally circular interference rings on the soap film were gradually reshaped into a predefined contour (for example, heart-shaped one). Guided by the non-uniform surface tension, the entire array was progressively transformed from its initial circular structure into a heart-shaped pattern.


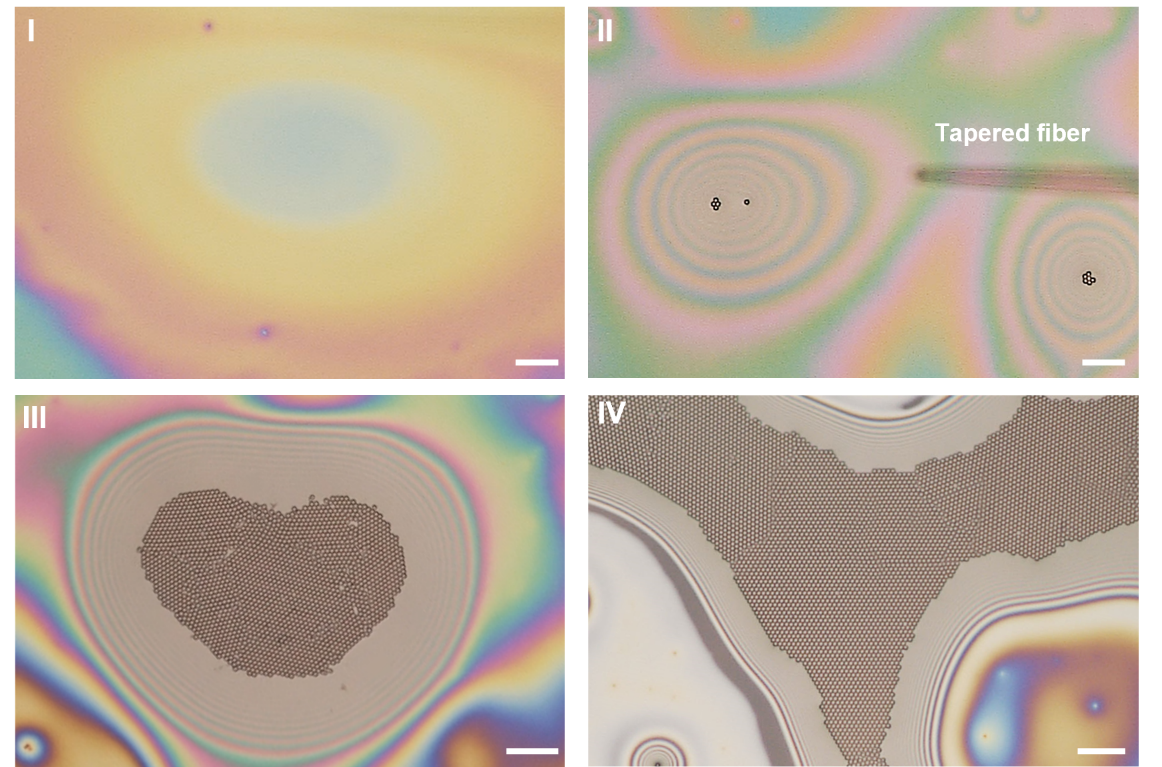


Fig. S1. (I) Bright-field image of a soap film. (II-IV) Microlens arrays assembled on the soap film via OTT method. Panels II-IV show microlens arrays with 10, 898, and 2036 microlenses assembled, respectively. Scale bar: 30 μm.

1. **Large-scale photonic nanojet effect via different microlenses**


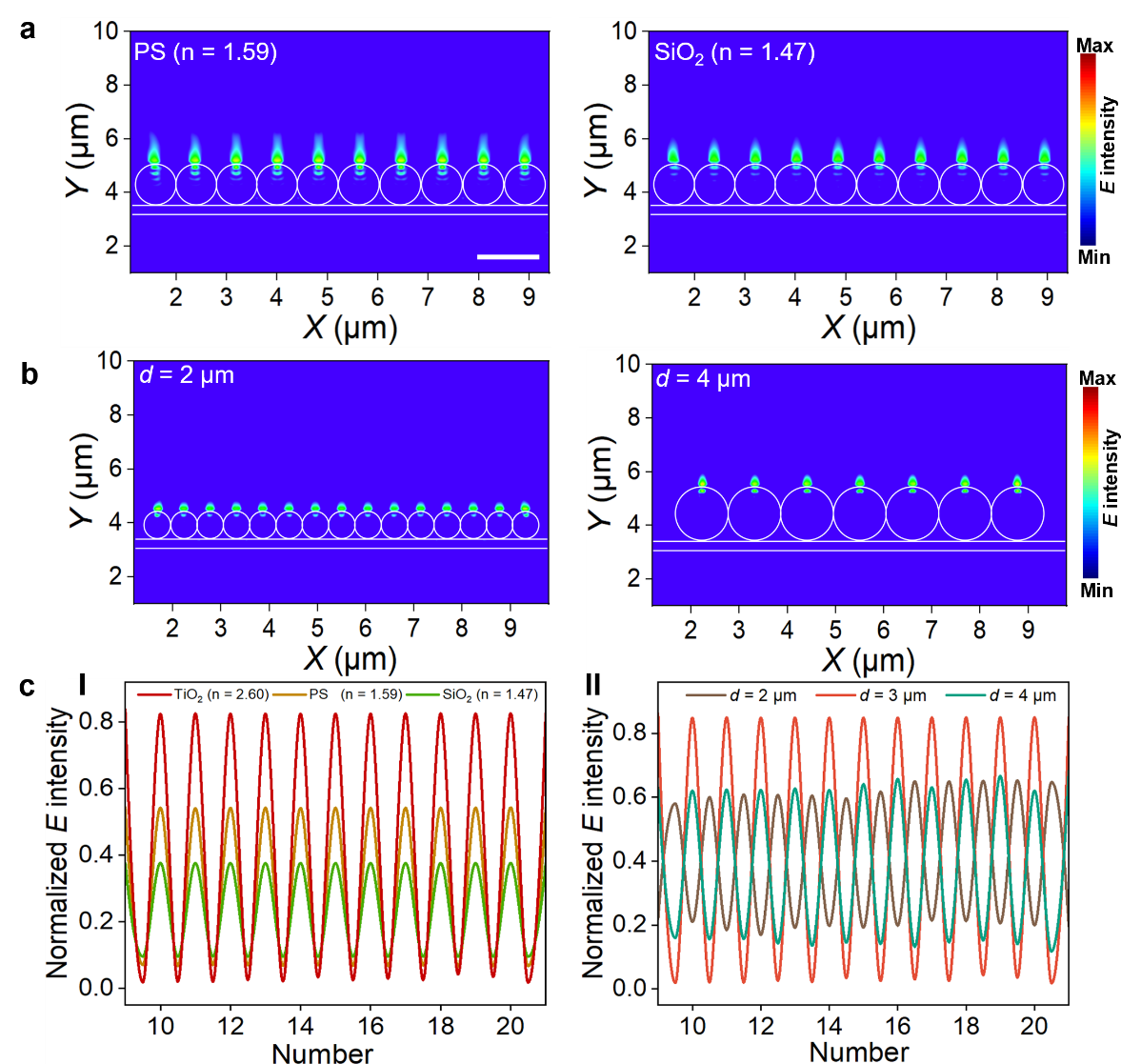


Fig. S2. Simulated light field distributions of microlens arrays. (a) Light field distribution of a microlens array constructed with 3-μm PS and SiO_2_. (b) Light field distribution of microlens arrays constructed with 2-μm and 4-μm TiO₂ microspheres. (c) Panel I: Normalized electric field intensity of microlens arrays composed of 3-μm TiO₂, PS and SiO_2_. Panel II: Normalized electric field intensity of TiO₂ microlens arrays with particle sizes of 2, 3, and 4 μm. Scale bar: 5 μm.


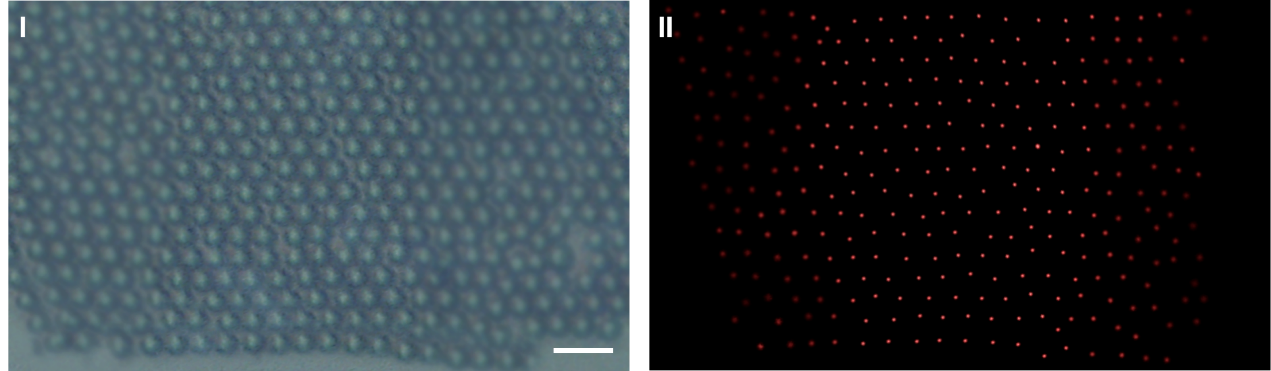


Fig. S3. Panel I: Bright-field image showing microlens array on a curved and stretched substrate. Panel II: Focused light spots of the microlens array on the curved and stretched substrate. Scale bar: 15 μm.

1. **Experimental setup**


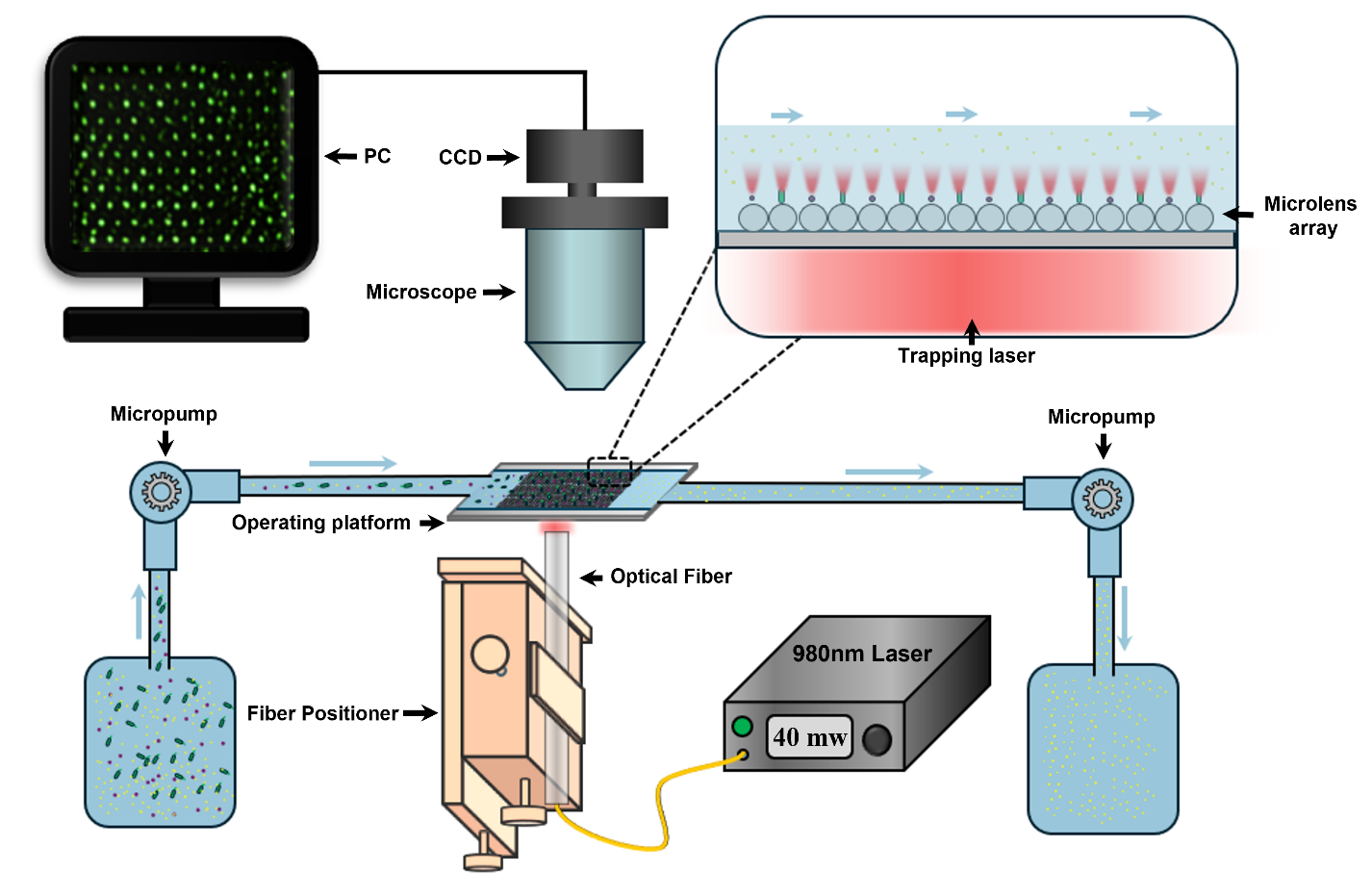


Fig. S4. Schematic diagram of the experimental setup. The FSOT was placed on a movable two-dimensional operation platform. The microflow with particles was introduced toward the FSOT via a micropump. The optical fiber probe was fixed on a five-axis adjustable micro-manipulator and was closely attached to the bottom of the FSOT. The 980-nm laser beam was launched to the optical fiber probe and was emitted onto the FSOT. Real-time images were recorded by a computer-interfaced high-speed CCD camera.

1. **High-throughput bioparticle trapping/separation on flat substrate**


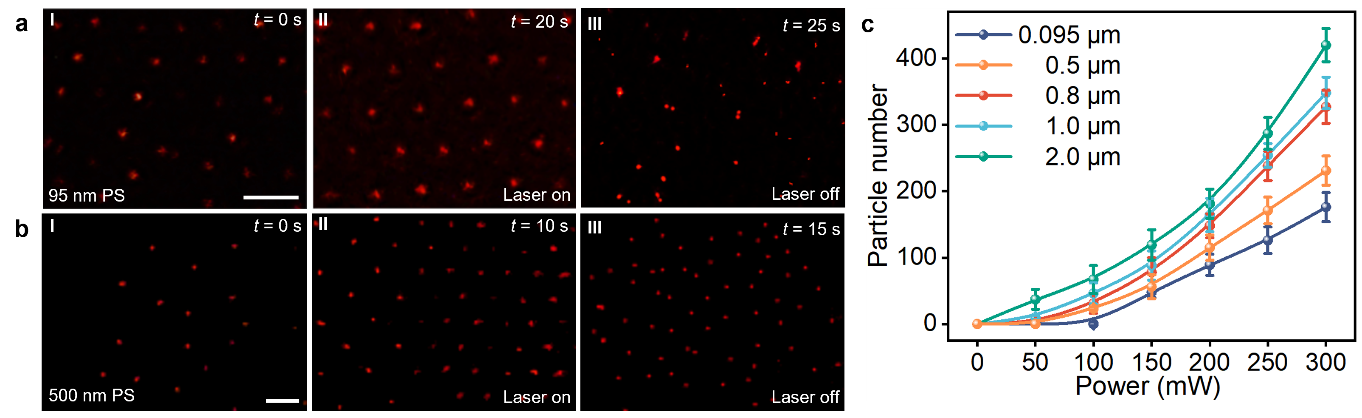


Fig. S5. (a, b) Trapping of 95nm and 500 nm PS microspheres. Panel III shows the particles were released when laser is off. (c) Number of trapped particles as a function of laser power for different sized particles. Scale bar: 3 μm.


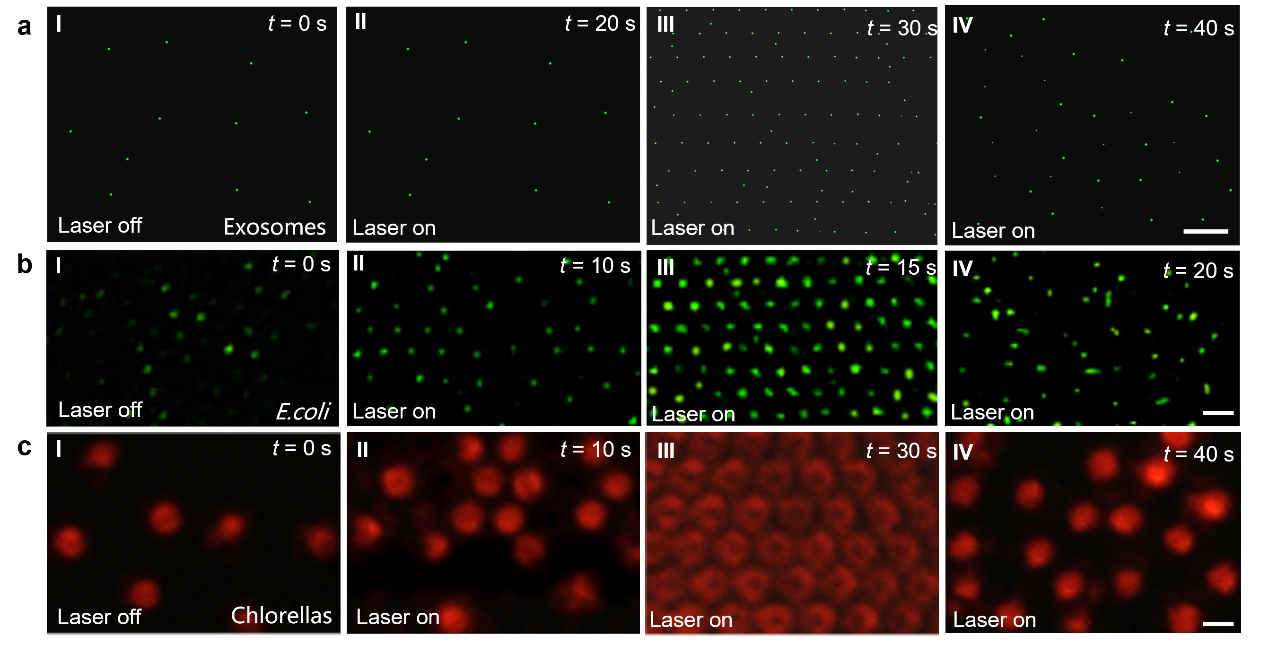


Fig. S6. High-throughput trapping of different bioparticles, (a) exosomes, (b) *E. coli*, and (c) Chlorella. Panels I-III are the trapping process, and panels IV show the release after laser off. Scale bar: 5 μm.


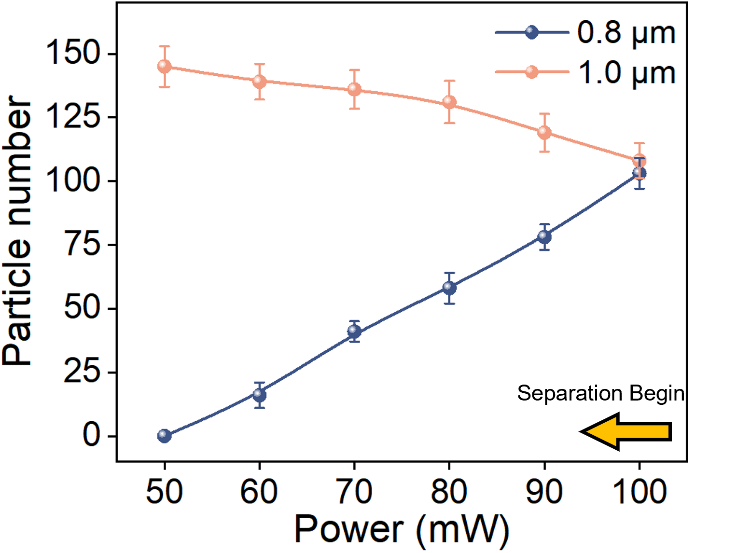


Fig. S7. Number of trapped particles as a function of laser power during particle sorting. Both particles were trapped with an initial power of 100 mW. As the laser power decreased from 100 to 40 mW, the 0.8-μm microspheres were gradually released from the FSOT, and both particles were totally sorted with the power decreased to 50 mW.

1. **High-throughput bioparticle trapping/separation on curved substrate**

The experiment was carried out on a flexible PDMS substrate with a length of 1 cm, a width of 1 cm and a thickness of 200 μm, which could be bent into a circular arc shape. The bending angle *θ* of the substrate is defined as the angle between the tangent at the free end and the horizontal direction. Based on geometric relationships, this angle is equal in magnitude to the central angle *θ* of the circular arc. Therefore, to achieve accurate bending in the experiments, the central angle was used to ensure that each bending was stable and repeatable. As shown in Fig. S8, on the bent substrate, the stationary end of the substrate is labeled as S, and the free end is labeled as F. The center of the circular arc formed after bending is labeled as O, with a radius of *r* and curvature *a*. When the flexible substrate was bent to 20°, 30°, and 40° respectively, the *r* was 2.86, 1.91, and 1.43 cm, and the curvatures *a* was 0.35, 0.52, and 0.70 rad cm^-1^. (Fig. S8).

Numerical calculations were performed to analyze the light field distributions of microlenses on substrates with different bending angles (Fig. S9). The results demonstrated that as the flexible FSOT system was bent progressively to 20°, 30°, and finally 40°, increased bending angles led to significantly larger local incidence angles, resulting in intensified Fresnel reflection losses and increased attenuation of transmitted light intensity. This reduced light intensity resulted in the decay of the trapping force.

Experimental results also validated this decreased trapping capability. As shown in Fig. S11a, when the FSOT was at a flat state (bending angle *θ* = 0°), about 200 PS particles (800 nm) were trapped. As shown in Fig.S11b, c, and d, when the flexible substrate was bent to 20°, 30°, and 40°, the number of stably trapped particles was decreased to 183, 143, and 72, respectively, due to the attenuation of the light field induced with increased bending angles. It should be noted that, in region *x* > 30 μm, the disparity in incidence angles caused by bending was more pronounced than that in *x* < 30 μm. Consequently, substrates with larger bending angles in *x* > 30 μm exhibit intensified Fresnel reflection losses, ultimately leading to a more significant attenuation of trapping forces. This was validated by the number of stable trapping particles of 79, 48, and 24 in the *x* > 30 μm region for 20°, 30°, and 40° bent substrates. Moreover, at the most curved end of the 40° bent substrate (where the bending angle reaches 40°), 800-nm particles could not be stably trapped. These results verified the simulations and confirm that the FSOT retained the trapping capabilities at the bending angles up to 40°.

Furthermore, the trapping stability of the FSOT at bending state was analyzed by calculating the mean-square displacement (MSD) of the trapped particles and the trapping stiffness *k*. On the 40° bent substrate, the positional data of all captured particles were recorded. Specifically, we extracted the positional variation data for particles located in the 45 < *x* < 60 region (Fig. S14a). The trapping stiffness *k* at different trapping positions was then calculated according to the MSD and the exerted optical force. Through MSD analysis, we quantified the *k* of optical traps generated by the FSOT at different trapping positions. As shown in Fig. S14b, *k* was decreased nonlinearly with the bending position. In the region *x* ≤ 30 μm, *k* remained stable at ~578 pN μm^-1^. The decline in the range is only 3%. In contrast, at the position of *x* = 60 μm, *k* was reduced to 177 pN μm^-1^ (about 30.6% reduction), and thus the particle trapping stability was significantly decreased due to the hydrodynamic or thermal disturbances. Therefore, the FSOT will lose the stable trapping ability.

These results indicate that our FSOT enables high-throughput particle trapping in environments with complex morphology and bent substrate, but the trapping stability is decreased with the increase in bending angle, and the trapping failed in highly curved regions (*θ* > 40°).


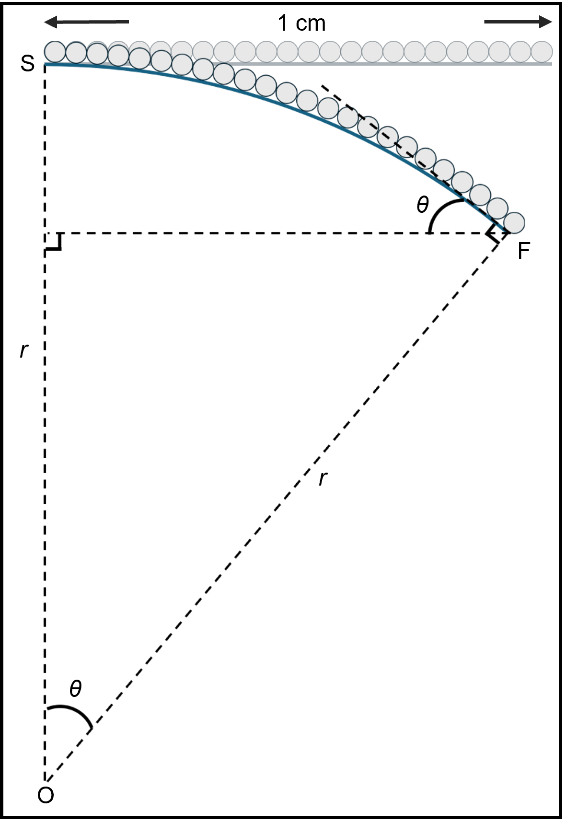


Fig. S8. Schematic diagram of the bent substrate. On the bent substrate, S, F, O, *r*, and *θ* indicate the stationary end of the substrate, the free end, the center of the circular arc, the radius of curvature, and the bending angle, respectively.


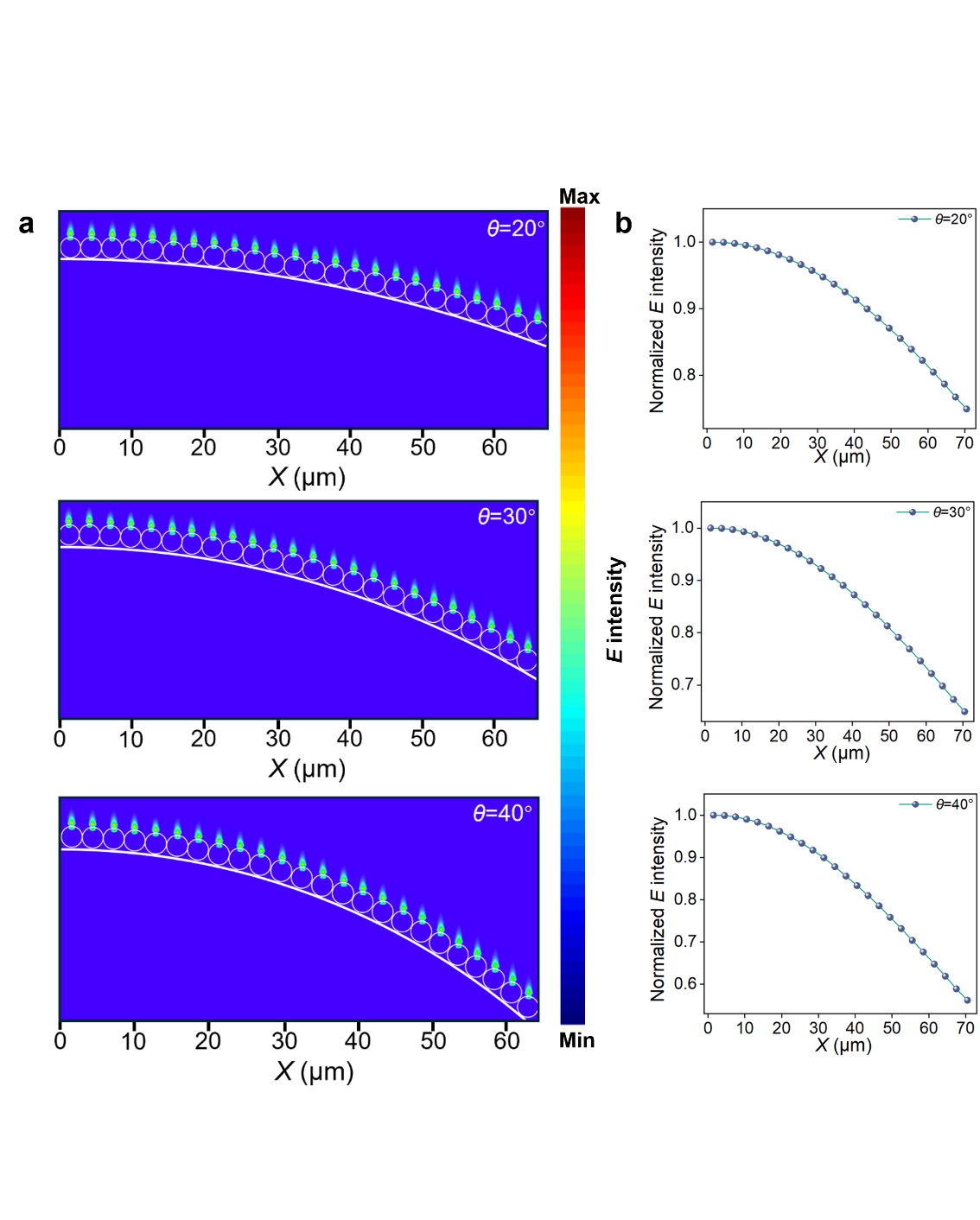


Fig. S9. (a) Simulated optical field distribution of microlens array on bent PDMS substrate. (b) Normalized optical field intensity on the top of the microlens array on substrates with different bending angles.

.
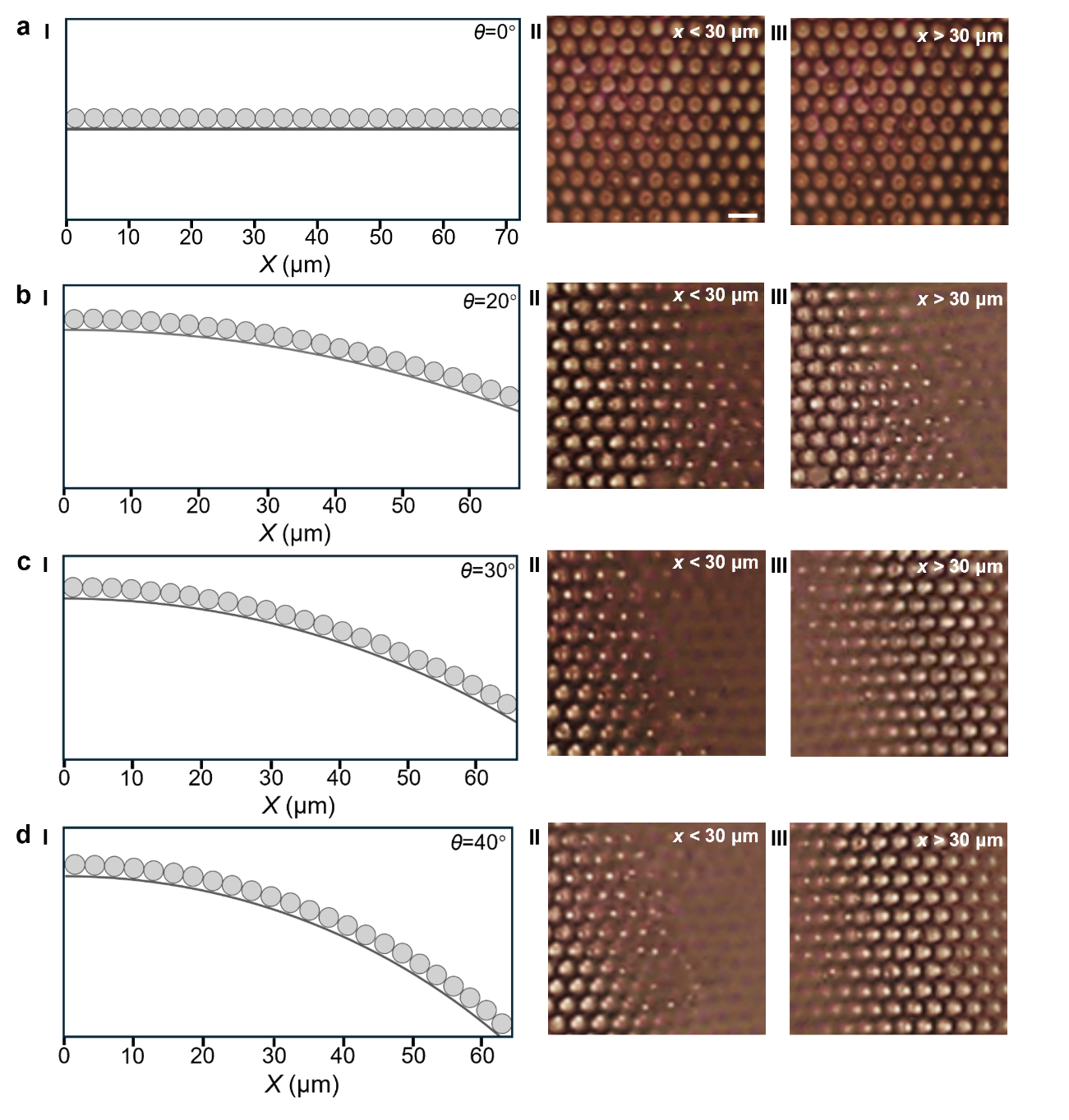


Fig. S10. Panel I: Position distribution of microlenses on (a) 0°, (b) 20°, (c) 30°, and (d) 40° bent FSOT. Panel II: Bright-field images of the trapping in the region (*x* < 30 μm) Panels III: Bright-field images of the trapping in the region (*x* >30 μm). Scale bar: 10 μm


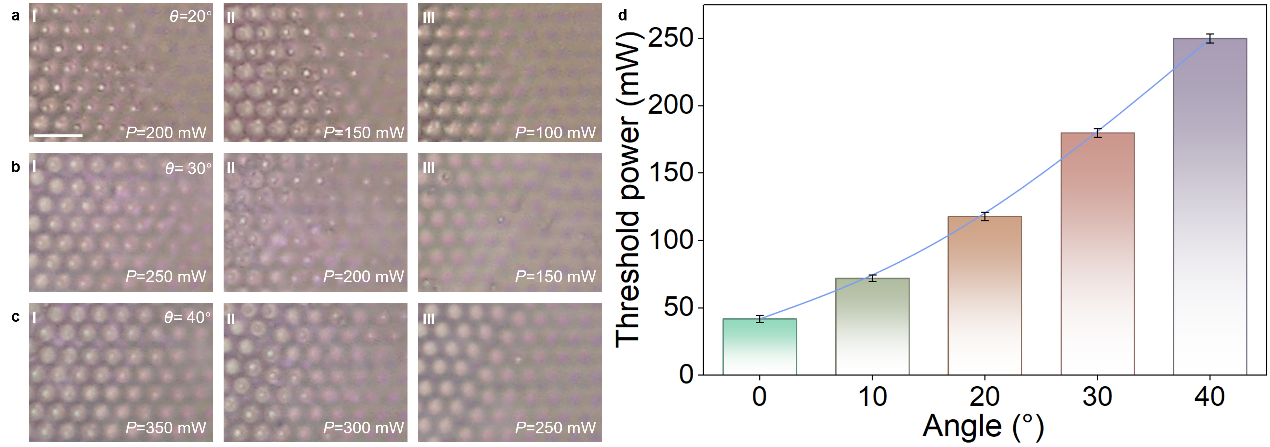


Fig. S11. Trapping with different bending angles. (a-c) Microscopic images showing the trapping of 1 μm PS particles using FSOT with substrates bent at (a) 20°, (b) 30°, and (c) 40°. (d) Trapping power threshold as a function of bending angle. Scale bar: 10 μm.


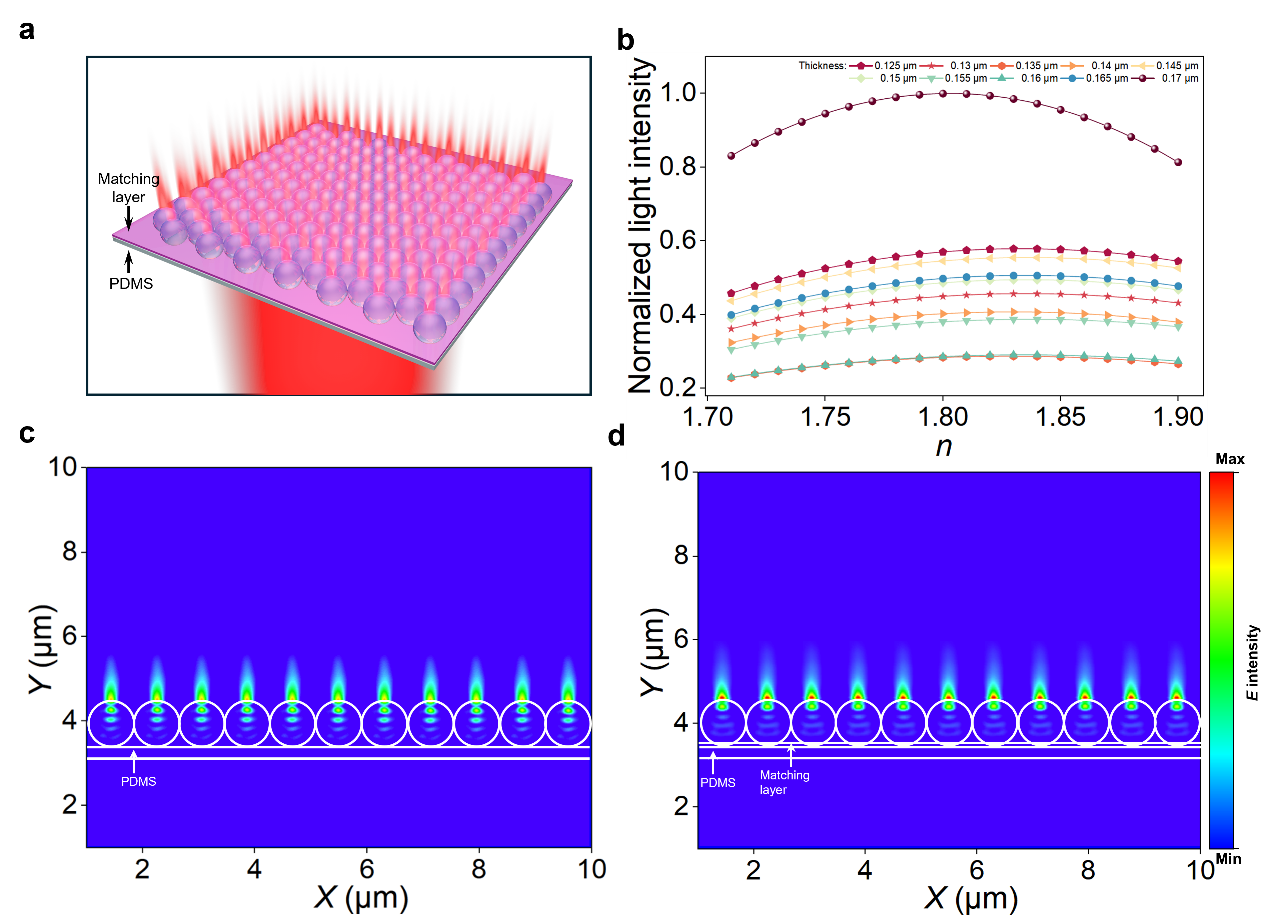


Fig. S12. (a) Schematic showing FSOT with a refractive index-matching layer between PDMS substrate and microlenses. (b) Normalized light field intensity at the top of 3-μm TiO2 microlense as a function of different refractive index and different thickness of the index-matching layer. (c-d) Simulated light field distribution after light propagating through the PDMS substrate and microlenses (c) without and (d) with the index-matching layer.


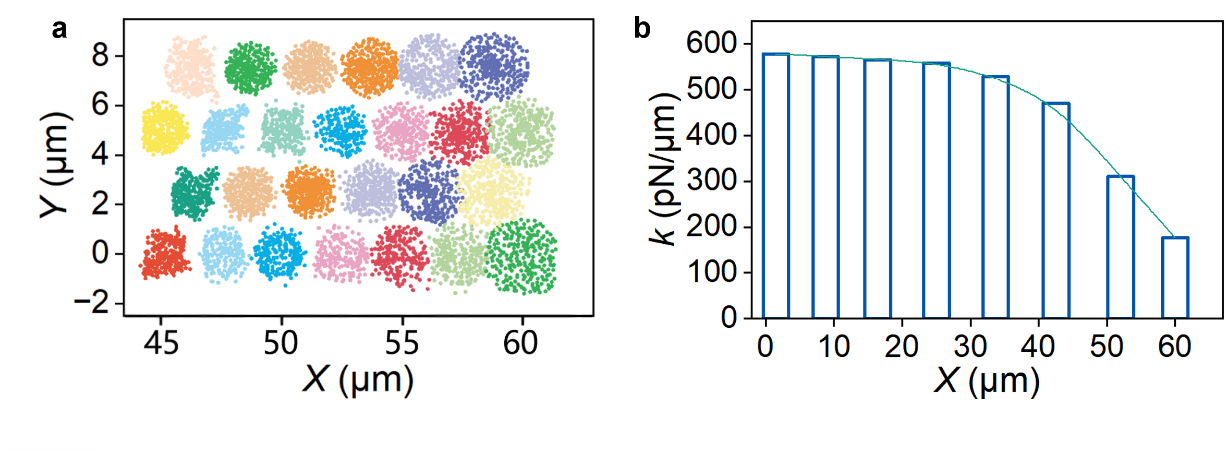


Fig. S13. (a) Position distribution of trapped 800-nm microspheres on 40° bent FSOT. (b) The stiffness *k* of the optical traps as a function of the microlens position along *x* direction.


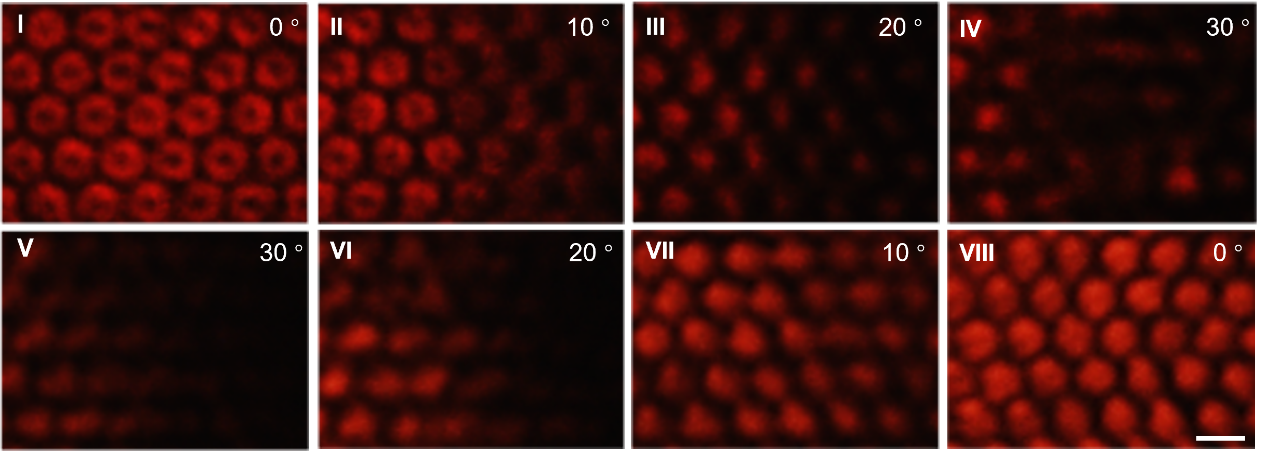


Fig. S14. Trapping of Chlorella on bent FSOT. Panels I-IV show the trapping of Chlorella during the FSOT was bent from 0° to 30°. Panels V-VIII show the trapping during the FSOT was recovered from 30° to 0°. Scale bar: 5 μm.


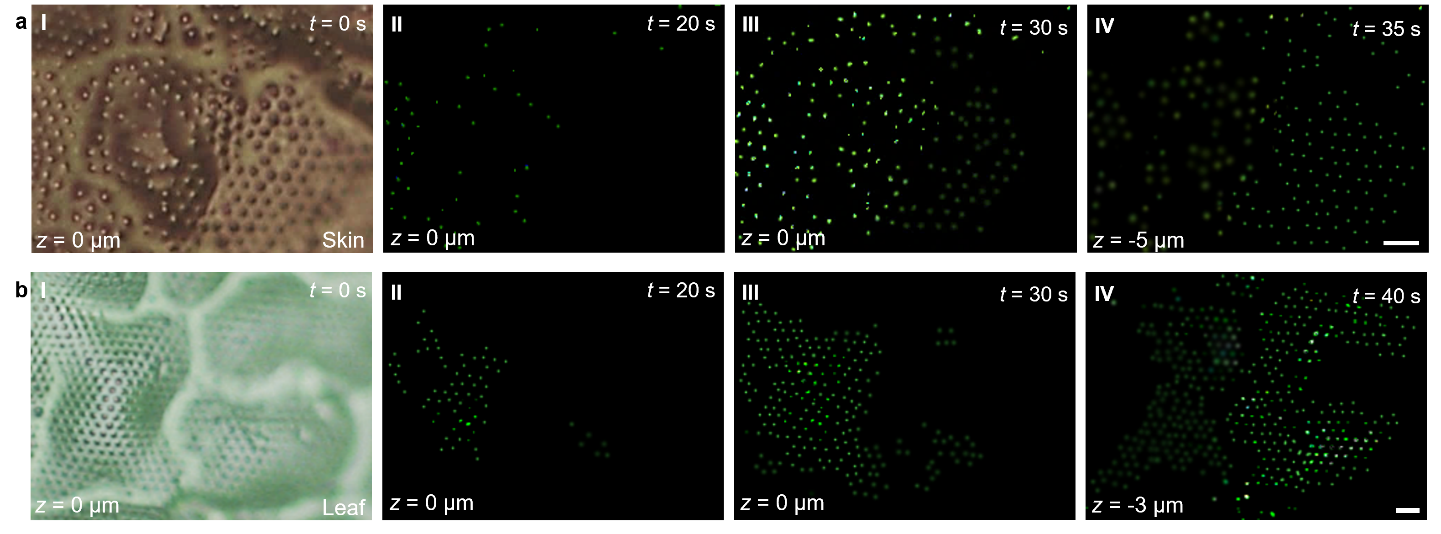


Fig. S15. Trapping of exosomes on curved biological substrates of (a) skin and (b) leaf. Scale bar: 10 μm.

To validate the long-term stability and trapping performance of the FSOT on living tissues environment, we first constructed the FSOT on sheep intestinal tissue, and then conducted a long-term *S. aureus* trapping experiment (120 minute, trapping power: 250 mW). The stability and trapping performance during prolonged operation were evaluated by analyzing morphological changes in the microlens array and the number of trapped bacteria during this period. As shown in Fig. S16, the overall morphology and tightly arranged structure of the microlens array remained stable after the 120-min trapping experiments. Also, with the extended laser irradiation (250 mW) on the FSOT assembled on the sheep intestinal tissues for 120 min, no obvious morphology changes were observed on the intestinal surface. These results indicate no obvious photothermal damage was observed to the tissues during the extend laser irradiation. To show the stable trapping of *S. aureus* during the long-term period, *S. aureus* was labeled with Live & Dead Bacterial Staining Kit (green for live bacteria). As shown in Fig.S17a, high-throughput trapping of *S. aureus* remained stable throughout the 120-minute experimental duration. No obvious decrease was observed on the trapped green *S. aureus* during the extended trapping process, indicating that the viability of the trapped bacteria was not significantly affected. The number of trapped bacteria consistently fluctuated slightly around approximately 250 (Fig.S17b), with no obvious decreasing trend. These results indicate that the microlens array of our FSOT on living tissues exhibits high stability during prolonged operation with the trapping performance effectively sustained.


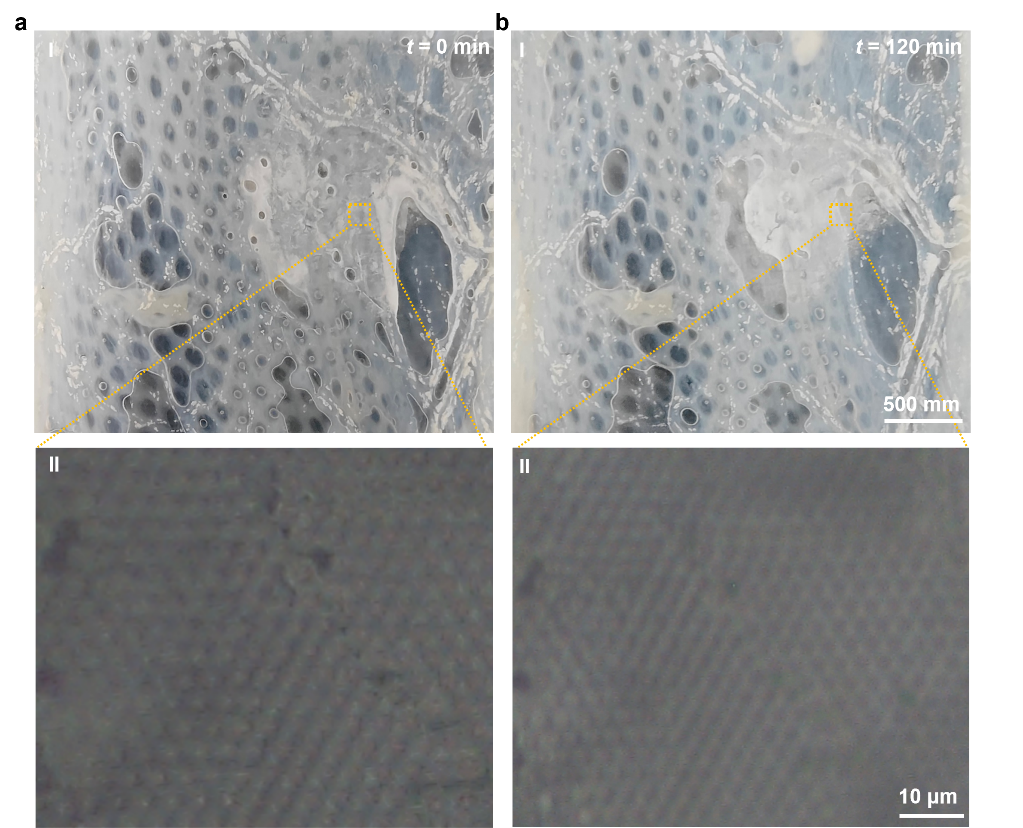


Fig. S16. (a-b) Panel I: Microscopic images showing the morphology of intestinal tissue with FSOT assembled (a) before and (b) after the 120-min trapping experiment. Panel II: Zoom-in microscopic images showing the microlens array (a) before and (b) after the 120-min trapping experiment.


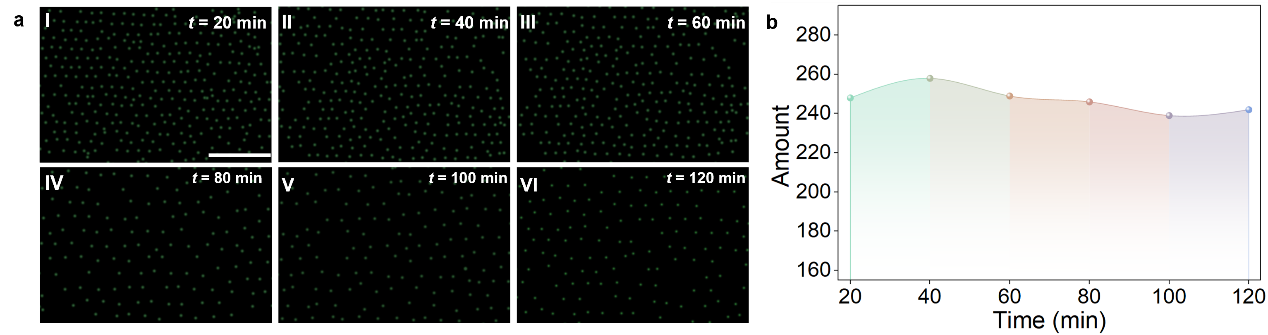


Fig. S17. (a) Fluorescence microscopic images showing the trapping of S. aureus (green fluorescence) within 120 min. Scale bar: 20 μm. (b) Number of trapped S. aureus as a function of time during the prolonged trapping experiments.


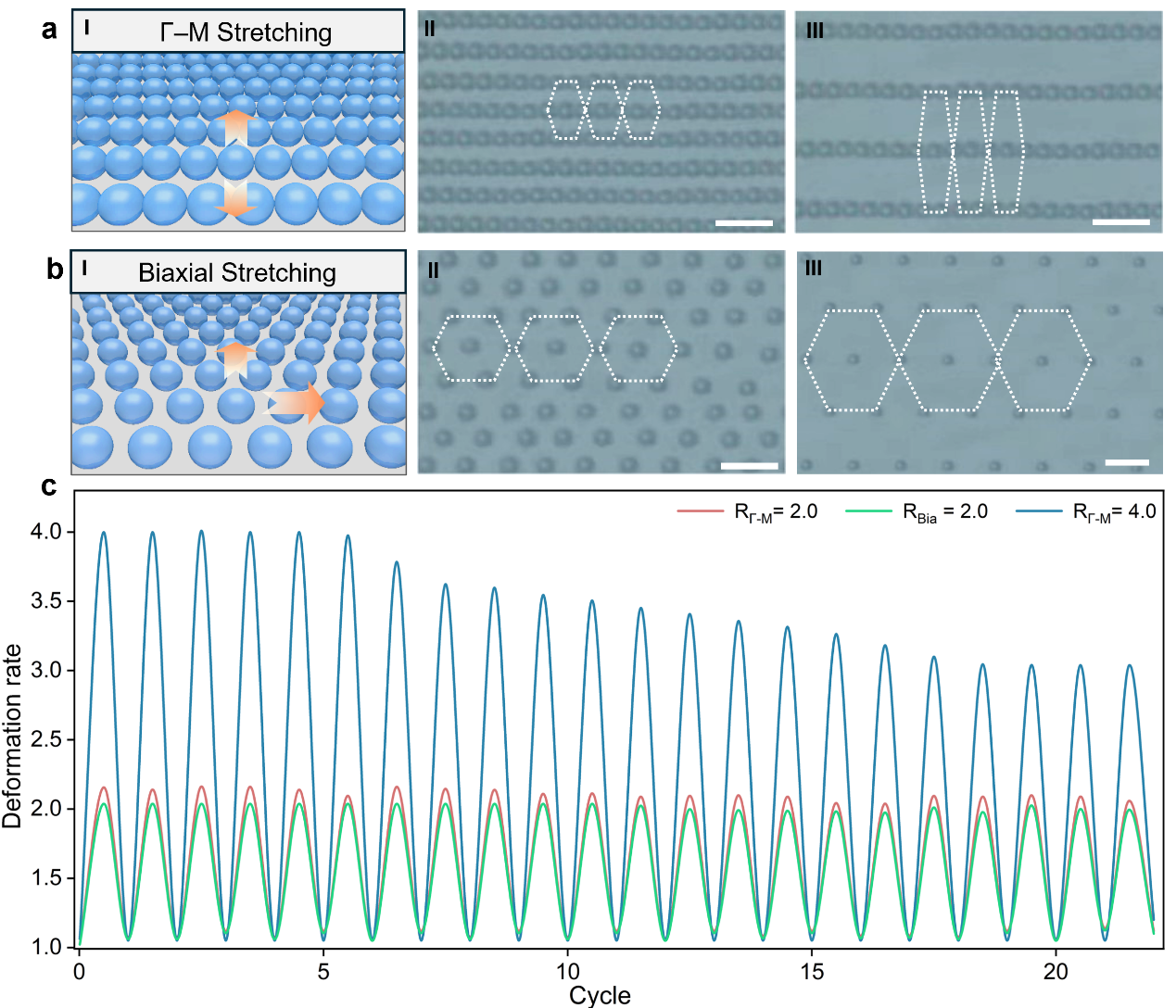


Fig. S18. Stretchability and recoverability of microlens array when stretched along different directions. (a,b) Stretching along (a) uniaxial (perpendicular) and (b) biaxial (both perpendicular and parallel) direction. Panel I: schematic showing the stretching. Panel II: stretching with deformation rate of 2.0. Panel III: stretching with deformation rate of 4.0. (c) Stretching recovery capacity of the microlens array as a function of deformation cycles. Scale bar: 10 μm.


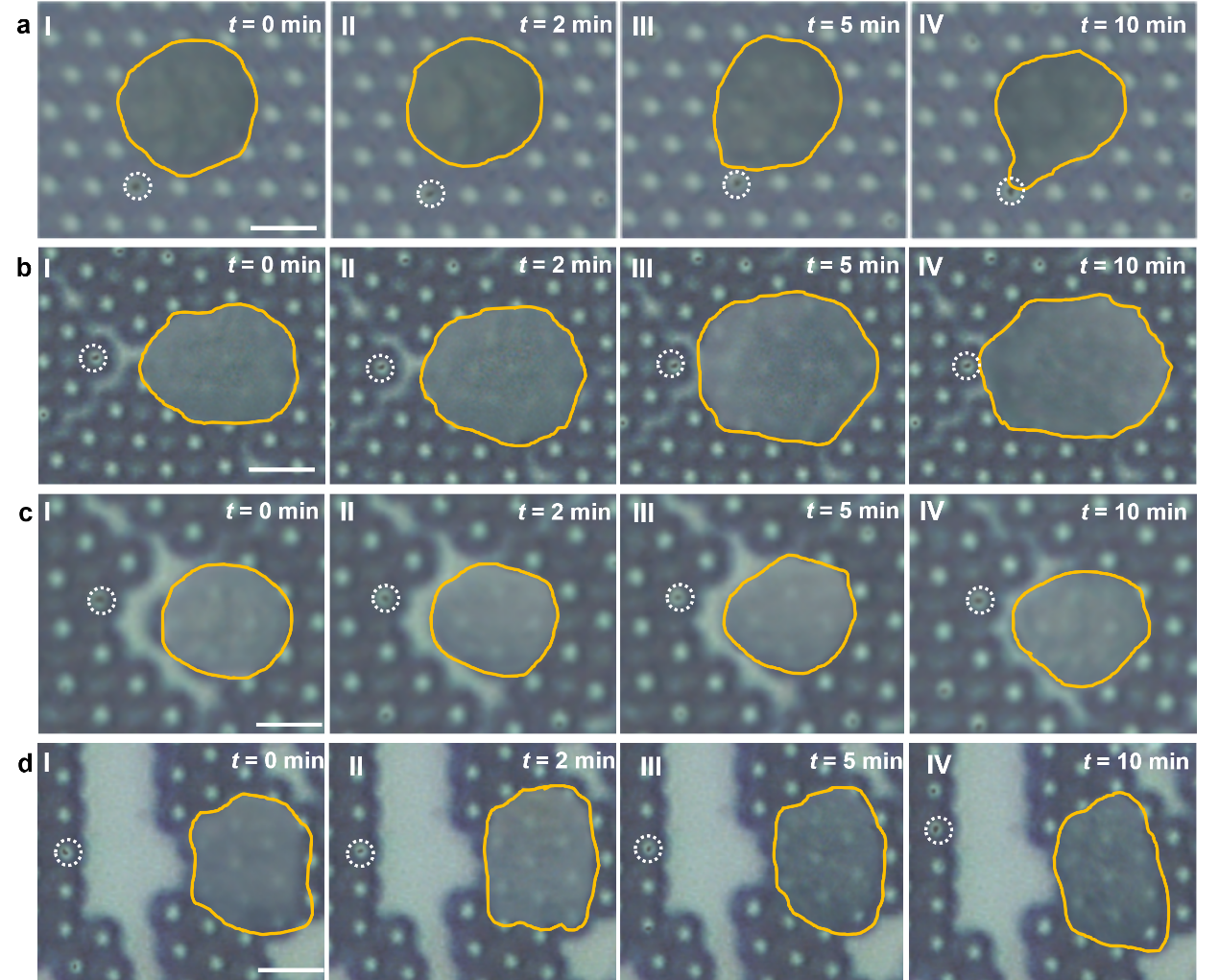


Fig. S19. Real-time monitoring of interactions between macrophages and *E. coli* at different initial interaction distances of (a) 1.0, (b) 1.5, (c) 3.0, and (d) 6.5 μm. The white curved dashed lines represent the positions of the trapped *E. coli*. The yellow curved lines represent the contours of the macrophages. Scale bar: 5 μm.


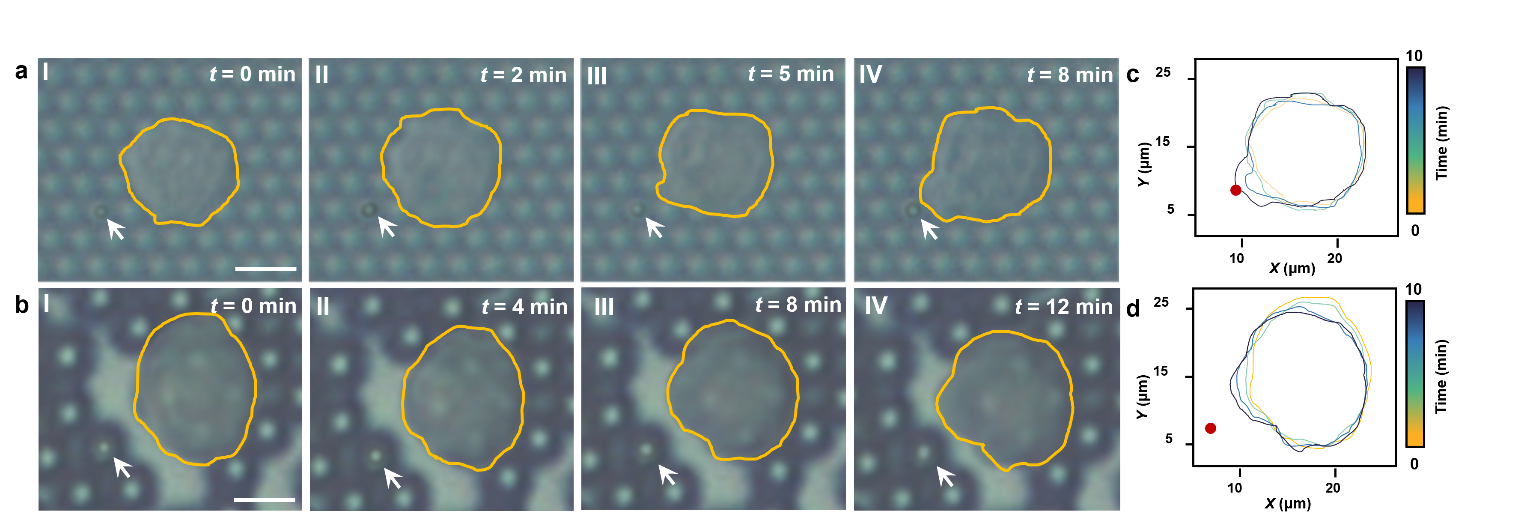


Fig. S20. (a, b) Real-time monitoring interactions between macrophages and *S. aureus* at different initial distances of (a) 1.0 and (b) 3.0 μm. The white arrows represent the positions of the trapped *S. aureus*. The yellow curved lines represent the contours of the macrophages. (c, d) Real-time cellular boundaries showing cellular response of macrophage to *S. aureus* at different initial distances of (c)1.0 and (d) 3.0 μm. The dot indicates the position of *S. aureus.* The color scale bar represents time. Scale bar: 5 μm.
